# Genome-Wide Analysis of Transcriptional Changes and Genes That Contribute to Fitness during Degradation of the Anthropogenic Pollutant Pentachlorophenol by Sphingobium chlorophenolicum

**DOI:** 10.1128/mSystems.00275-18

**Published:** 2018-11-20

**Authors:** Jake J. Flood, Shelley D. Copley

**Affiliations:** a Department of Molecular, Cellular and Developmental Biology, University of Colorado Boulder, Boulder, Colorado, USA; b Cooperative Institute for Environmental Sciences, University of Colorado Boulder, Boulder, Colorado, USA; Leiden University

**Keywords:** RNA-seq, *Sphingobium chlorophenolicum*, Tn-seq, benzoquinone, biodegradation, hydroquinone, pentachlorophenol

## Abstract

Pentachlorophenol (PCP) is a highly toxic pesticide that was first introduced in the 1930s. The alphaproteobacterium Sphingobium chlorophenolicum, which was isolated from PCP-contaminated sediment, has assembled a metabolic pathway capable of completely degrading PCP. This pathway produces four toxic intermediates, including a chlorinated benzoquinone that is a potent alkylating agent and three chlorinated hydroquinones that react with O_2_ to produce reactive oxygen species (ROS). RNA-seq analysis revealed that PCP causes a global stress response that resembles responses to proton motive force uncoupling and membrane disruption, while surprisingly, little of the response resembles the responses expected to be produced by the PCP degradation intermediates. Tn-seq was used to identify genes important for fitness in the presence of PCP. By comparing the genes that are important for fitness in wild-type S. chlorophenolicum and a non-PCP-degrading mutant, we identified genes that are important only when the PCP degradation intermediates are produced. These include genes encoding two enzymes that are likely to be involved in protection against ROS. In addition to these enzymes, the endogenous levels of other enzymes that protect cells from oxidative stress appear to mitigate the toxic effects of the chlorinated benzoquinone and hydroquinone metabolites of PCP. The combination of RNA-seq and Tn-seq results identify important mechanisms for defense against the toxicity of PCP.

**IMPORTANCE** Phenolic compounds such as pentachlorophenol (PCP), triclosan, and 2,4-dichlorophenoxyacetic acid (2,4-D) represent a common class of anthropogenic biocides. Despite the novelty of these compounds, many can be degraded by microbes isolated from contaminated sites. However, degradation of this class of chemicals often generates toxic intermediates, which may contribute to their recalcitrance to biodegradation. We have addressed the stresses associated with degradation of PCP by Sphingobium chlorophenolicum by examining the transcriptional response after PCP exposure and identifying genes necessary for growth during both exposure to and degradation of PCP. This work identifies some of the mechanisms that protect cells from this toxic compound and facilitate its degradation. This information could be used to engineer strains capable of improved biodegradation of PCP or similar phenolic pollutants.

## Abstract (precis)

Phenolic compounds such as pentachlorophenol (PCP), triclosan, and 2,4-dichlorophenoxyacetic acid (2,4-D) represent a common class of anthropogenic biocides. Despite the novelty of these compounds, many can be degraded by microbes isolated from contaminated sites. However, degradation of this class of chemicals often generates toxic intermediates, which may contribute to their recalcitrance to biodegradation. We have addressed the stresses associated with degradation of PCP by Sphingobium chlorophenolicum by examining the transcriptional response after PCP exposure and identifying genes necessary for growth during both exposure to and degradation of PCP. This work identifies some of the mechanisms that protect cells from this toxic compound and facilitate its degradation. This information could be used to engineer strains capable of improved biodegradation of PCP or similar phenolic pollutants.

## INTRODUCTION

Pentachlorophenol (PCP) is listed as a priority pollutant by the U.S. Environmental Protection Agency and is banned by the Stockholm Convention on Persistent Organic Pollutants. It is currently used in the United States as a wood preservative, and it is used more widely in China to kill snails that transmit schistosomiasis (1, 2). Concern about its use has arisen due to its inherent toxicity (3), recalcitrance (4, 5), and potential for long-range dispersal in the environment (6, 7). PCP is an endocrine disruptor (8) and a potential carcinogen (9). Alarmingly, it has recently been found in human fluid and tissue samples (10) as well as in the food chain (11).

Many microbes are capable of degrading PCP (12); the best characterized is Sphingobium chlorophenolicum, an alphaproteobacterium that mineralizes PCP (13–15) and has been used in studies aimed at improving PCP biodegradation (16–19). Degradation of PCP, which was introduced into the environment in the 1930s (20), is interesting from both an evolutionary and practical standpoint. Emergence of a new pathway for degradation of an anthropogenic compound requires recruitment of previously existing enzymes with at least a modest ability to convert the novel compound into a familiar metabolite that can be degraded by existing pathways. Not all microbes will have suitable promiscuous enzymes available to assemble a new pathway. The challenge of evolving a new pathway extends beyond recruitment of new enzymes, however. When a new pathway is introduced into a metabolic network, new metabolites may be toxic, either inherently or because they interfere with the functions of proteins that have not evolved to exclude previously unseen metabolites from sensitive sites (21).

Understanding the stresses caused by novel intermediates is also relevant for efforts to improve biodegradation, which often focus on improving flux through the pathway of interest by directed enzyme evolution (22, 23), tuning expression levels (24), and engineering posttranscriptional control (25). Understanding the stresses produced by the novel pathway and the defense mechanisms that contribute to survival may reveal additional strategies for improving biodegradation (26).

**FIG 1:**
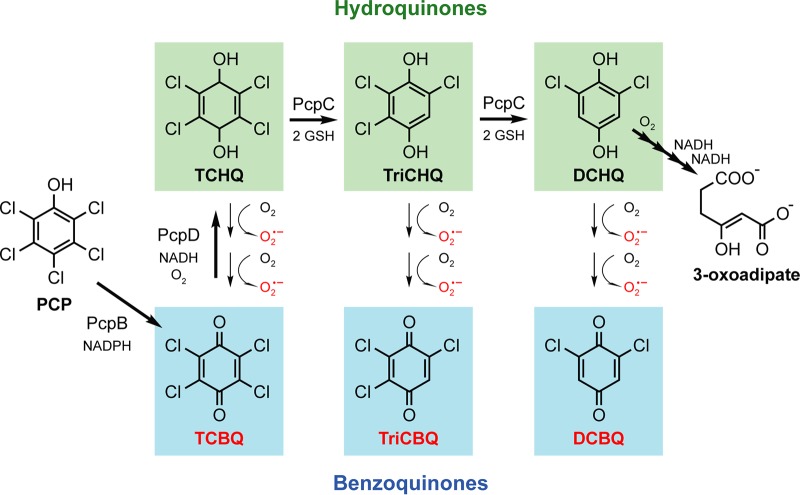
Degradation of PCP by S. chlorophenolicum and nonenzymatic oxidation of degradation intermediates. PcpB is PCP hydroxylase, PcpD is TCBQ reductase, and PcpC is TCHQ dehalogenase. Steps not shown include those catalyzed by PcpA (2,6-dichlorohydroquinone dioxygenase) and PcpE (maleylacetate reductase). PCP, pentachlorophenol; TCBQ, tetrachlorobenzoquinone; TCHQ, tetrachlorohydroquinone; TriCHQ, 2,5,6-trichlorohydroquinone; DCHQ, 2,6-dichlorohydroquinone; TriCBQ, 2,5,6-trichlorobenzoquinone; DCBQ, 2,6-dichlorobenzoquinone; GSH, glutathione.

S. chlorophenolicum faces a number of stresses during PCP degradation. The toxicity of PCP itself has been attributed to disruption of the cell envelope (27–31) and dissipation of the proton motive force (PMF) (32–34). PCP is converted to tetrachlorobenzoquinone (TCBQ) by PcpB (Fig. 1) (15). TCBQ is highly toxic (LD_50_ of <1 µM in Escherichia coli [35]) because it reacts rapidly with nucleophiles, such as those found in glutathione, proteins, and DNA (36–38). PcpD reduces TCBQ to tetrachlorohydroquinone (TCHQ) while it is still within the active site of PcpB (39). TCHQ then undergoes reductive dehalogenation to 2,5,6-trichlorohydroquinone (TriCHQ) and then to 2,6-dichlorohydroquinone (DiCHQ); both reactions are catalyzed by PcpC (40, 41).

Highly reactive benzoquinone and hydroquinone intermediates also occur during biodegradation of other widely used substituted chloro- and nitrophenols (42–45). 2,4,5-Trichlorophenol and 2,4-dichlorophenol are breakdown products of the pesticides 2,4,5-trichlorophenoxyacetic acid (2,4,5-T) and 2,4-dichlorophenoxyacetic acid (2,4-D), respectively. Nitrophenols are used in the production of pesticides such as parathion and nitrophen. Thus, problematic intermediates are a common problem during degradation of anthropogenic phenols.

The toxicity of PCP and its metabolites begs the question of how S. chlorophenolicum deals with the stresses associated with PCP degradation. The concentrations of chlorinated hydroquinone intermediates in cells during PCP degradation sum to approximately 61 µM (35). Exposure of HepG2 cells to 10 µM TCHQ produces significant amounts of reactive oxygen species (ROS) and decreases mitochondrial membrane potential (46). Exposure of primary splenocytes to 12.5 µM TCHQ produces ROS and substantially decreases viability (47). Treatment of hamster lung fibroblasts with 25 µM TCHQ for 1 h increases the level of 8-hydroxy-2-oxoguanosine, a marker of oxidative DNA damage, and induces single-strand breaks (48). Thus, the levels of the chlorinated hydroquinone metabolites during PCP degradation are in the range that causes toxic effects in mammalian cells. S. chlorophenolicum may be more or less capable of preventing ROS production and damage to macromolecules.

To explore the specific stresses perceived by S. chlorophenolicum during PCP degradation, we identified differentially expressed genes after exposure to PCP as well as a variety of other stressors using RNA-seq. The transcriptional response to PCP shared substantial overlap with responses to toluene and carbonyl cyanide *m*-chlorophenyl hydrazone (CCCP), which cause membrane disruption and dissipation of the PMF, respectively, but little overlap with responses to methylglyoxal and paraquat, which cause alkylation and ROS production, respectively. These results suggest that the majority of the transcriptional response during PCP degradation is caused by PCP itself and that S. chlorophenolicum largely avoids stresses caused by degradation intermediates, possibly because transcription of genes involved in defense against the downstream metabolites is already sufficient.

We identified genes that are important for fitness during PCP degradation using transposon sequencing (Tn-seq). Libraries in which every nonessential gene in the genome was disrupted by insertion of a transposon were grown for 20 to 25 generations in the presence of 200 µM PCP. We identified 76 genes whose disruption changes fitness in the presence of PCP but not in its absence. Notably, far fewer genes are important for fitness than change in expression in the presence of PCP, suggesting that much of the transcriptional response is wasteful. Genes encoding components of efflux pumps and membrane remodeling proteins are particularly important, suggesting that removal of PCP from the cell and membrane adaptation are important mechanisms of resistance to PCP toxicity. Interestingly, four genes are no longer important for fitness when PCP degradation is prevented by deletion of *pcpB*, which encodes the first enzyme in PCP degradation. Three of these genes may contribute to protection against the toxic degradation intermediates.

## RESULTS

### PCP degradation both detoxifies the medium and provides a novel carbon source.

**FIG 2:**
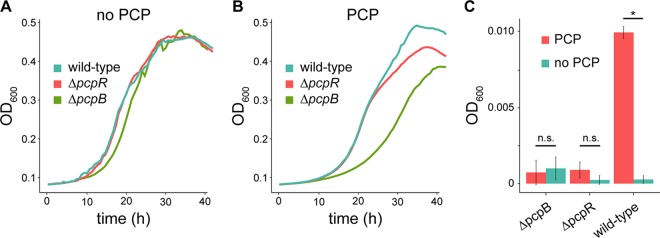
Disruption of PCP degradation impacts growth in the presence of PCP. Representative growth curves for the wild-type, Δ*pcpR*, and Δ*pcpB* strains in SCD medium in the absence (A) or presence (B) of 200 µM PCP. (C) Final OD_600_ of three replicate cultures of wild-type, Δ*pcpR*, and Δ*pcpB* strains of S. chlorophenolicum grown in minimal salts medium (initial OD_600_ of 0.001) in the presence or absence of 200 µM PCP after 8 days incubation at 30˚C with shaking. *, *P* value of <0.001; n.s., not significant.

Although PCP degradation involves exposure to PCP and a number of toxic metabolites, it must benefit the organism. We examined the growth of wild-type, Δ*pcpR,* and Δ*pcpB* S. chlorophenolicum strains in the presence and absence of 200 µM PCP (Fig. 2A and B). (PcpR is the transcriptional activator for the PCP degradation genes.) We observed a 10% and 23% decrease in biomass yield of the Δ*pcpR* and Δ*pcpB* strains, respectively, compared to the biomass yield of the wild-type strain in the presence of PCP. Under these growth conditions, the wild-type strain depletes PCP from the medium, while the mutant strains endure constant PCP stress, but no stress from the metabolites. Not surprisingly, detoxification of the environment by PCP degradation is beneficial. However, this may not be the only benefit of degrading PCP. PCP could be used as a carbon source, although a relatively poor one because it is already highly oxidized.

To test whether PCP can serve as a carbon source, starter cultures of wild-type, Δ*pcpR*, and Δ*pcpB* S. chlorophenolicum were inoculated into minimal salts medium in the presence or absence of 200 µM PCP. The wild-type strain grew 10-fold in the presence of 200 µM PCP, whereas neither mutant grew (Fig. 2C), demonstrating that S. chlorophenolicum can use PCP as a source of carbon and energy.

### PCP exposure causes a massive transcriptional response.

Given that PCP degradation is beneficial to S. chlorophenolicum despite the myriad challenges posed by PCP and its metabolites, we were intrigued by the question of how the bacterium responds to and manages the stresses of PCP degradation. We assessed the transcriptional response of S. chlorophenolicum after exposure to 200 µM PCP for 15 min and 5 h by RNA-seq (see Data Set S1A in the supplemental material). Short-read sequencing was carried out on RNA isolated from triplicate samples (see Fig. S1A to C in the supplemental material), yielding 3 to 10 million reads per replicate. After 15 min of PCP exposure, 479 genes were upregulated and 364 were downregulated by >2-fold. After 5 h of PCP exposure, the transcriptional response was reduced; only 312 genes were upregulated, and 51 were downregulated by >2-fold. Thus, 19.9% and 8.6% of the genome was differentially expressed by >2-fold after 15-min or 5-h exposure to PCP, respectively. RNA-seq results correlated well (*R*^2^ = 0.938) with RT-qPCR measurements of the effects of PCP exposure on expression of 16 representative genes (Fig. S2).

10.1128/mSystems.00275-18.1 FIG S1 Correlation of results for replicate samples from S. chlorophenolicum after growth in 1/4-strength tryptic soy broth in the absence of PCP (A) and after exposure to 200 µM PCP for 15 min (B), 200 µM PCP for 5 h (C), 39 µM CCCP for 15 min (D), 5 mM toluene for 15 min (E), 20 µM paraquat for 15 min (F), or 1 mM methylglyoxal for 15 min (G). Mapped reads were filtered to remove reads from weakly expressed genes and noninformative reads. Pearson correlation coefficients were calculated for each set of replicates. Download FIG S1, JPG file, 1.2 MB. Copyright © 2018 Flood and Copley. 2018 Flood and Copley This content is distributed under the terms of the Creative Commons Attribution 4.0 International license.

10.1128/mSystems.00275-18.2 FIG S2 Validation of RNA-seq results by RT-qPCR. (A) Comparison of changes in expression after exposure to 200 µM PCP for 15 min relative to the control for individual genes as determined by RT-qPCR and RNA-seq. Relative expression was calculated with the Pfaffl method. (B) qPCR results correlate linearly with RNA-seq results. Download FIG S2, JPG file, 0.3 MB. Copyright © 2018 Flood and Copley. 2018 Flood and Copley This content is distributed under the terms of the Creative Commons Attribution 4.0 International license.

10.1128/mSystems.00275-18.10 DATA SET S1 (A) RNA-seq analysis of S. chlorophenolicum exposed to PCP, CCCP, toluene, paraquat, and methylglyoxal. (B) Tn-seq analysis of wild-type and Δ*pcpB* S. chlorophenolicum strains grown in the presence of 200 µM PCP. (C) Genes conditionally important during PCP exposure in the wild-type strain. (D) Genes conditionally important during PCP degradation (wild-type versus Δ*pcpB* strains). (E) Primers used in this study. Download Data Set S1, XLSX file, 1.9 MB. Copyright © 2018 Flood and Copley. 2018 Flood and Copley This content is distributed under the terms of the Creative Commons Attribution 4.0 International license.

**FIG 3:**
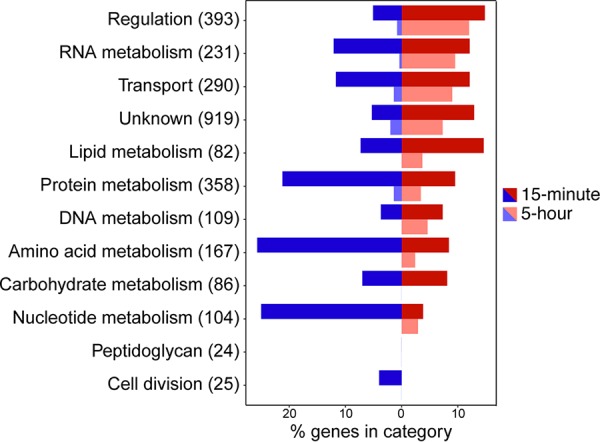
Exposure to PCP results in a substantial transcriptional response. Genes differentially expressed by >2-fold (false-discovery rate [FDR] of <0.01) after exposure to PCP for 15 min or 5 h were sorted according to their most relevant GO categories. The number of genes in each category of interest is labeled.

Differentially expressed genes were separated into categories of interest based on gene ontology (GO) annotations (Fig. 3). PCP exposure causes initial downregulation of genes involved in lipid, amino acid, carbohydrate, and nucleotide metabolism, as well as many genes involved in protein synthesis and turnover. Genes encoding classic stress response proteins such as GroEL, DnaK, and RpoH were initially upregulated but returned to normal levels after 5 h. Overall, expression of 76% of differentially regulated genes normalized between 15 min and 5 h of PCP exposure, even though the level of PCP in the medium remained unchanged. These data suggest that physiological adaptations quiet the initial global transcriptional response. However, many genes, particularly those involved in transcription, gene regulation, and transport, remain differentially expressed after 5 h. One hundred sixty-three genes are differentially expressed only at the 5-h time point (Fig. S3A).

10.1128/mSystems.00275-18.3 FIG S3 Comparison of differentially expressed genes after exposure to 200 µM PCP for 5 h and other stressors for 15 min, including 200 µM PCP (A), 5 mM toluene (B), 39 µM CCCP (C), 20 µM paraquat (D), or 1 mM methylglyoxal (E). Spearman rank correlation coefficients were calculated using all significantly differentially expressed genes (FDR of <0.01). Download FIG S3, JPG file, 0.6 MB. Copyright © 2018 Flood and Copley. 2018 Flood and Copley This content is distributed under the terms of the Creative Commons Attribution 4.0 International license.

*pcpR,* which encodes the PCP-responsive transcriptional regulator (49), and all PCP degradation genes except *pcpC* were strongly upregulated at both time points (Table 1)*. pcpC* was previously reported to be constitutively expressed (50). However, we observed that it was upregulated 3.3-fold after 5 h of exposure to PCP. The genes encoding PcaIJ and PcaF, which convert 3-oxoadipate, the end product of PCP degradation, to acetyl-CoA and succinyl-CoA, were upregulated by twofold only after 5 h, suggesting that production of 3-oxoadipate lags behind initiation of PCP degradation. This lag may be due to the low catalytic activity of the first enzyme in the pathway, PcpB, which has a *k*_cat_ of only 0.02 s^−1^ (38).

**TABLE 1:** Differential expression of genes of interest after PCP exposure^*a*^

Gene	Product	log_2_ Δ expression (15 min)	FDR	log_2_ Δ expression (5 h)	FDR
*pcpR*	PCP transcriptional activator	3.21*	2.9e−116	3.90*	1.2e−173
*pcpB*	PCP hydroxylase	6.89*	0.0e+0	8.91*	0.0e+00
*pcpD*	TCBQ reductase	5.40*	0.0e+0	7.04*	0.0e+00
*pcpC*	TCHQ dehalogenase	0.17	1.4e−01	1.74*	5.8e−65
*pcpA*	DCHQ dioxygenase	5.65*	1.8e−80	7.32*	1.1e−124
*pcpE*	Maleylacetate reductase	4.47*	2.9e−242	5.96*	0.0e+00
*pcaJ*	3-Oxoacid CoA transferase, B subunit	0.34	1.3e−03	1.02*	7.9e−26
*pcaI*	3-Oxoacid CoA transferase, A subunit	−0.13	3.5e−01	1.01*	2.2e−17
*pcaF*	Beta-ketoadipyl CoA thiolase	0.05	6.6e−01	0.90	7.4e−16
*gshA*	Glutamate-cysteine ligase	2.83*	1.5e−180	1.24*	2.3e−35
*gshB*	Glutathione synthetase	1.56*	6.2e−53	0.42	1.9e−04
*emrR*	TetR family transcriptional regulator	5.16*	5.3e−308	3.88*	1.9e−140
*emrA*	Efflux pump membrane protein	4.73*	4.0e−292	3.29*	3.2e−107
*emrB*	Drug resistance transporter	4.63*	0.0e+0	3.27*	1.2e−207
*fad1*	Fatty acid desaturase	2.31*	1.4e−109	0.50	6.8e−06
*fadD1*	Long-chain fatty acid-CoA ligase	1.19*	9.2e−24	−0.09	7.1e−01
*fadD2*	Long-chain fatty acid-CoA ligase	2.81*	3.1e−108	1.73*	3.9e−29
*fadD3*	Long-chain fatty acid-CoA ligase	4.82*	5.3e−270	1.38*	1.8e−17
*fadJ*	3-Hydroxybutyryl-CoA epimerase	1.67*	7.4e−75	0.61	3.1e−10
*fadH1*	2,4-Dienoyl-CoA reductase	2.72*	3.5e−62	0.59	4.2e−02
*mdoG*	Periplasmic glucan biosynthesis protein	2.34*	5.3e−84	0.50	4.6e−04
*dgkA*	Diacylglycerol kinase	2.58*	3.9e−113	0.54	1.5e−04

a All differentially expressed genes are listed in Data Set S1A in the supplemental material. This table contains genes that are discussed in the text. Genes whose expression changes significantly after PCP exposure, determined by a fold change of >2 and a false-discovery rate (FDR) of <0.01. Asterisks indicate significant differences.

Expression of glutathione biosynthesis genes (*gshA* and *gshB*) increased after PCP exposure. Glutathione serves many functions (51), including acting as a cosubstrate for PcpC (Fig. 1) and detoxifying ROS and benzoquinones generated during PCP degradation. Increased expression of *gshA* and *gshB* may protect cells against stresses caused by PCP degradation intermediates.

Genes encoding 14 different efflux pumps were upregulated after 15 min. Notable among these is EmrAB, which in E. coli confers resistance to hydrophobic toxins such as CCCP and nalidixic acid (52). Some of these pumps may facilitate removal of PCP or downstream intermediates from the cell.

PCP exposure also results in upregulation of many genes encoding enzymes involved in fatty acid metabolism, including fatty acid desaturase, three long-chain fatty acid-CoA ligases, 3-hydroxybutyryl-CoA epimerase, and 2,4-dienoyl-CoA reductase. Additionally, genes encoding a periplasmic glucan biosynthesis protein and diacylglycerol kinase are strongly upregulated; both have been implicated in the response to osmotic stress (53). These enzymes may help compensate for disruption of membrane fluidity caused by accumulation of PCP in the lipid bilayer.

### Tn-seq reveals genes that are important for fitness during growth in PCP.

**FIG 4:**
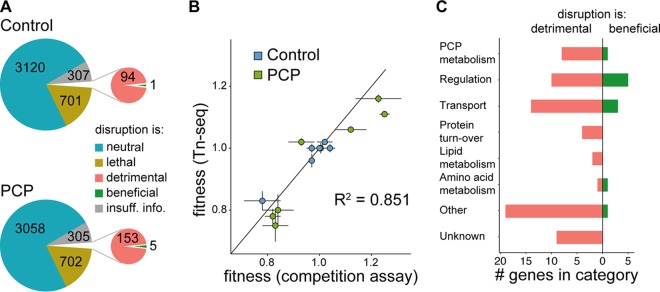
Identification of genes that are important for growth in the presence and absence of 200 µM PCP. (A) Genes sorted into categories based on the effect of disruption: detrimental (fitness < 0.95); beneficial (fitness > 1.05); neutral (fitness 0.95 to 1.05). Disruption of genes longer than 400 base pairs for which fewer than four insertions were observed in the library is apparently lethal. Insufficient information was available to assign categories for short genes (<400 base pairs) with fewer than four insertions. (B) Correlation between fitness determined by Tn-seq and direct competition between individual mutants and wild-type S. chlorophenolicum. (C) Mutants for which fitness was changed in the presence of 200 µM PCP but not in its absence sorted into functional categories.

Many genes that are part of a global stress response may be unimportant for fitness during PCP exposure; conversely, expression of some genes that are critical during PCP degradation may not change (54). To identify genes that are important specifically during PCP degradation, we used Tn-seq, which measures the relative fitness of thousands of clones in a genome-wide transposon insertion library (55). We created a saturated transposon library using the *Himar1C* mariner transposase (56). This library was grown in the presence or absence of 200 µM PCP for 20 to 25 generations. Genomic DNA was purified, and the regions surrounding the transposons were sequenced to identify their locations. Each sequenced library contained >3.6 million mapped reads after quality control filtering. We identified 66,026 unique transposon insertion sites (87% of possible insertion sites). The insertion density averaged 1 insert per 56 bp (Fig. S4A); each gene contained an average of 8.5 inserts. Comparing the relative frequency of reads for each insertion site before and after the outgrowth allowed us to calculate the fitness effect of disrupting each gene with good reproducibility between replicates (Fig. S5A and B). Disruption of genes that increased fitness (equation 2 in Text S1) by >0.05 was considered beneficial, while disruption of genes that decreased fitness by >0.05 was considered detrimental (Fig. 4A and Data Set S1B). One-to-one growth competition experiments between selected mutants and wild-type S. chlorophenolicum confirmed the fitness effects observed by Tn-seq (Fig. 4B and Table S1).

10.1128/mSystems.00275-18.4 FIG S4 (A) S. chlorophenolicum transposon library insert density as quantified by high-throughput sequencing. Arrow, average insertion density (1 insert per 56 base pairs). (B) Plasmid map of pSAM_Sc. Abbreviations: *ampR*, ampicillin resistance; *hygR*, hygromycin resistance; oriR6K, plasmid R6K origin of replication; oriT_RP4, origin of conjugative transfer RP4. Download FIG S4, JPG file, 0.3 MB. Copyright © 2018 Flood and Copley. 2018 Flood and Copley This content is distributed under the terms of the Creative Commons Attribution 4.0 International license.

10.1128/mSystems.00275-18.5 FIG S5 Correlation of fitness values between Tn-seq replicate libraries for wild-type and *ΔpcpB* S. chlorophenolicum strains grown in SCD medium for 20 to 25 generations in the absence of PCP (A and C) or in the presence of 200 µM PCP (B and D). Pearson correlation coefficients were calculated for each set of replicates. Download FIG S5, JPG file, 1.2 MB. Copyright © 2018 Flood and Copley. 2018 Flood and Copley This content is distributed under the terms of the Creative Commons Attribution 4.0 International license.

10.1128/mSystems.00275-18.8 TEXT S1 Supplemental methods. Download Text S1, PDF file, 0.1 MB. Copyright © 2018 Flood and Copley. 2018 Flood and Copley This content is distributed under the terms of the Creative Commons Attribution 4.0 International license.

10.1128/mSystems.00275-18.9 TABLE S1 Validation of Tn-Seq results by growth competition assays. Download Table S1, PDF file, 0.02 MB. Copyright © 2018 Flood and Copley. 2018 Flood and Copley This content is distributed under the terms of the Creative Commons Attribution 4.0 International license.

We identified 71 and 5 genes for which disruption was detrimental or beneficial, respectively, in the presence of 200 µM PCP but neutral in its absence (Data Set S1C). Figure 4C shows the GO categories to which these genes belong. Disruption of genes encoding PCP degradation enzymes was detrimental, even though the medium was never detoxified because the PCP concentration was held constant during the experiment. Clones lacking any of the downstream enzymes would accumulate toxic intermediates, so this decrease in fitness is expected. The observation that disruption of *pcpB*, which encodes the first enzyme in the pathway, was also detrimental suggests that an intact PCP degradation pathway mitigates the toxic effects of PCP, possibly by diminishing the concentration of PCP within the cell. Surprisingly, disruption of *pcpR*, which encodes the PCP transcriptional activator, was beneficial. The reason for the discrepancy between the effects of disrupting *pcpR* and *pcpB,* which in both cases prevents PCP degradation, will be addressed in the Discussion.

Disruption of genes encoding two three-component efflux pumps, including an uncharacterized RND family efflux transporter and EmrAB, was detrimental. Disruption of *emrR*, which encodes the transcriptional regulator for EmrAB, was beneficial, suggesting that EmrR is a transcriptional repressor. These findings are consistent with a previous report that mutations in *emrR* in E. coli lead to increased expression of *emrA/emrB* and increased resistance to protonophores (57). The importance of these efflux pumps suggests that the amount of PCP that can enter the cell exceeds the capacity of the degradation pathway to remove it and consequently causes toxicity.

Disruption of several regulatory proteins, including two sensor histidine kinases and eight transcriptional regulators, was detrimental. These proteins, in addition to PcpR, are likely to orchestrate important components of the transcriptional response to PCP, although their specific roles are unknown.

Interestingly, a comparison of the Tn-seq and RNA-seq results revealed little correlation between the change in fitness upon disruption of a gene and the change in its expression (Fig. S6), suggesting that most of the transcriptional response to PCP is wasteful. Some of the transcriptional response is even counterproductive; expression of some genes that are important for fitness during PCP degradation is actually decreased in response to PCP and vice versa. Much of this wasteful and counterproductive response to PCP may be the result of the general stress response. This phenomenon has been observed in many other studies (54, 58–60).

10.1128/mSystems.00275-18.6 FIG S6 Fitness of transposon integrants does not correlate with changes in expression of the corresponding genes after PCP exposure. Expression fold change after a 15-min (A) or 5-h (B) exposure to PCP versus fitness (*W*) difference of transposon integrants in the presence and absence of PCP. Changes in gene expression that are consistent with the importance of the gene for fitness in the presence of PCP are considered appropriate (blue). Changes in gene expression that are opposite those expected based upon the importance of the gene for fitness in the presence of PCP are considered counterproductive (red). The size of each point is determined by the *P* value associated with the fitness difference calculation. Download FIG S6, JPG file, 0.6 MB. Copyright © 2018 Flood and Copley. 2018 Flood and Copley This content is distributed under the terms of the Creative Commons Attribution 4.0 International license.

### Comparative RNA-seq reveals a majority of the stress response is due to PCP itself rather than its degradation intermediates.

To identify which stresses caused by PCP and its metabolites are responsible for transcriptional changes during PCP degradation, we performed RNA-seq after exposing S. chlorophenolicum to one of four model stressors—CCCP, toluene, methylglyoxal, or paraquat—for 15 min. CCCP, methylglyoxal, and paraquat were used at concentrations that, like 200 µM PCP, slightly reduce the growth rate of S. chlorophenolicum (Fig. S7), thus elucidating a transcriptional response without killing a large portion of the population. S. chlorophenolicum proved to be resistant to high concentrations of toluene, so a concentration that had previously been shown to elucidate a transcriptional response in Pseudomonas putida was used (61). All RNA-seq experiments were performed in biological triplicate (Fig. S1D to G) and resulted in 3 to 12 million reads per experiment.

10.1128/mSystems.00275-18.7 FIG S7 Inhibition of S. chlorophenolicum growth in 1/4-strength tryptic soy broth by chosen concentrations of various stressors. Growth curves are representative of four replicates. Download FIG S7, JPG file, 0.4 MB. Copyright © 2018 Flood and Copley. 2018 Flood and Copley This content is distributed under the terms of the Creative Commons Attribution 4.0 International license.

**FIG 5:**
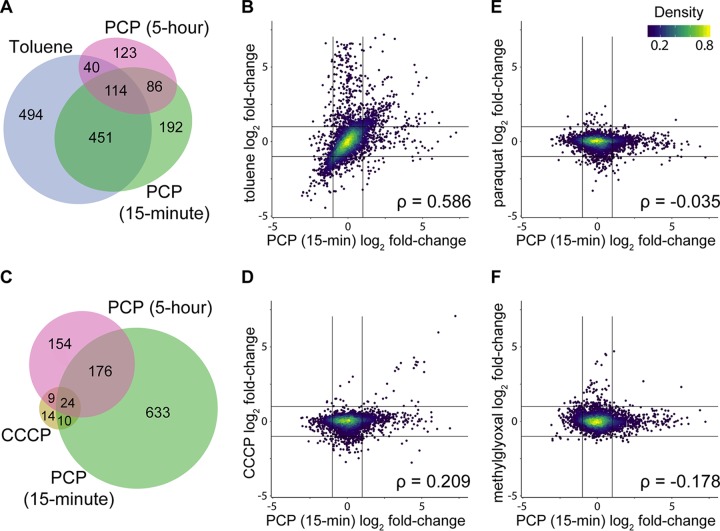
Comparison of differentially expressed genes after exposure to 200 µM PCP and other stressors, including 5 mM toluene (A and B), 39 µM CCCP (C and D), 20 µM paraquat (E), or 1 mM methylglyoxal (F). Spearman rank correlation coefficients were calculated using all significantly differentially expressed genes (FDR < 0.01).

To determine which aspects of the transcriptional response are associated with accumulation of hydrophobic molecules in the membrane, we measured the response of S. chlorophenolicum to toluene. Exposure to 5 mM toluene induced a massive transcriptional response; expression of 1,099 genes was altered by >2-fold (Data Set S1A). Many of these genes are involved in phospholipid/cell wall metabolism or cell envelope stress. Many classic stress response genes are also upregulated after toluene exposure. The responses to toluene and PCP strongly overlap (Spearman correlation coefficient of 0.586) (Fig. 5A and B). These results suggest that the majority of the transcriptional response to PCP is caused by its impact on the cell membrane. Less overlap is seen between the responses to 15-min toluene exposure and 5-h PCP exposure (Spearman correlation coefficient of 0.376) (Fig. 5B and Fig. S3B), suggesting that the impact of membrane disruption by PCP has been largely mitigated by 5 h.

The toxicity of PCP itself has been primarily attributed to its ability to dissipate the PMF (32–34). Maintaining the PMF is critical, as it is required for many cellular processes, including the activity of ATP synthase and RND and MFS family efflux pumps (62). To determine how much of the transcriptional response to PCP is due to dissipation of the PMF, we measured the response of S. chlorophenolicum to the protonophore CCCP. After 15-min exposure to CCCP, expression of 57 genes was altered by >2-fold (Data Set S1A). These include genes encoding proteins in the electron transport chain and several efflux pumps. The majority of genes that are strongly differentially expressed after CCCP exposure also change in response to 15-min (Fig. 5C and D) or 5-h (Fig. S3C) exposure to PCP. These data suggest that exposure of S. chlorophenolicum to PCP causes dissipation of the PMF akin to that caused by CCCP.

To determine whether production of superoxide by oxidation of the chlorinated hydroquinone intermediates contributes to the transcriptional response to PCP, we measured the transcriptional response after exposure to paraquat (63). The response was subtle; expression of 29 genes was altered by >2-fold (Data Set S1A). Several genes involved in defense against ROS were upregulated; these include genes encoding catalase-peroxidase (2.2-fold), alkyl hydroperoxide reductase (3.2-fold), ferredoxin-NADP^+^ reductase (2.2-fold), and NAD(P)^+^ transhydrogenase (*pntAB*) (1.6 and 2-fold). However, we saw little overlap with the transcriptional response after 15-min (Fig. 5E) or 5-h (Fig. S3D) PCP exposure (Spearman correlation coefficients of −0.035 and 0.059, respectively).

To determine whether alkylation of macromolecules contributes to the transcriptional response to PCP, we measured the transcriptional response after exposure to methylglyoxal, a toxic electrophile (64). Expression of 118 genes was altered by >2-fold (Data Set S1A). Genes involved in DNA repair changed in expression; *lexA* and *radA* were upregulated by 4.9- and 4.2-fold, respectively. Several genes involved in glutathione-dependent detoxification of methylglyoxal were also upregulated. The transcriptional responses to methylglyoxal and either 15-min (Fig. 5F) or 5-h (Fig. S3E) exposure to PCP showed little correlation (Spearman correlation coefficients of −0.178 and −0.013, respectively).

This comparison of transcriptional responses reveals that 4.8% of the transcriptional response to PCP is shared with the response to dissipation of the PMF, while 67% is shared with the response to disruption of the cell membrane, suggesting that a majority of the stress associated with PCP exposure is due to its impact on the cell envelope. Little of the response is due to stresses produced by ROS or alkylating agents. These findings suggest that constitutive defense mechanisms are sufficient to handle the stresses caused by the intermediates.

### PCP exposure dissipates the PMF but does not impact [ATP]/[ADP].

**FIG 6:**
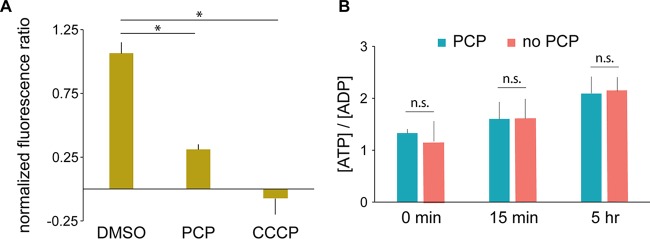
PCP affects the PMF but not [ATP]/[ADP]. (A) Effect of PCP or CCCP on intracellular pH as measured by the ratiometric pH-dependent fluorescent probe BCECF-AM. *, *P* value of <0.006. (B) [ATP]/[ADP] in S. chlorophenolicum cultures. All *P* values are >0.5 (not significant [n.s.]).

The correlation between the transcriptional responses of CCCP and PCP suggests that disruption of the PMF is one of the largest stresses imposed on S. chlorophenolicum during PCP degradation. Indeed, past reports have primarily attributed the toxicity of PCP in various cell types to its ability to act as a protonophore (32–34). To determine whether S. chlorophenolicum maintains any PMF under the conditions used in these studies, we measured the effect of PCP on intracellular pH using a ratiometric pH-dependent fluorescent probe, 2′,7′-bis-(2-carboxyethyl)-5-(and-6)-carboxyfluorescein acetoxymethyl ester (BCECF-AM). After the addition of 200 µM PCP, fluorescence immediately decreased by 71% (Fig. 6A), indicating that the intracellular pH had dropped dramatically due to dissipation of the PMF, although not as severely as the drop after the addition of 39 µM CCCP, which decreased the intracellular pH to the external pH (7.1). We conclude that the PMF is largely but not completely dissipated upon addition of 200 µM PCP. Therefore, PMF-driven transporters may be able to function, though perhaps not at full capacity. Remarkably, the ratio of [ATP] to [ADP] was not affected by the addition of 200 µM PCP, although it increased somewhat over time in both the control and PCP-treated samples (Fig. 6B). Since ATP synthase relies on the PMF, this finding suggests that S. chlorophenolicum is either able to maintain the activity of ATP synthase despite a compromised PMF or to fall back on aerobic fermentation to supplement ATP synthesis in the presence of 200 µM PCP.

### Comparative Tn-seq reveals genes that are important during PCP degradation rather than just PCP exposure.

**FIG 7:**
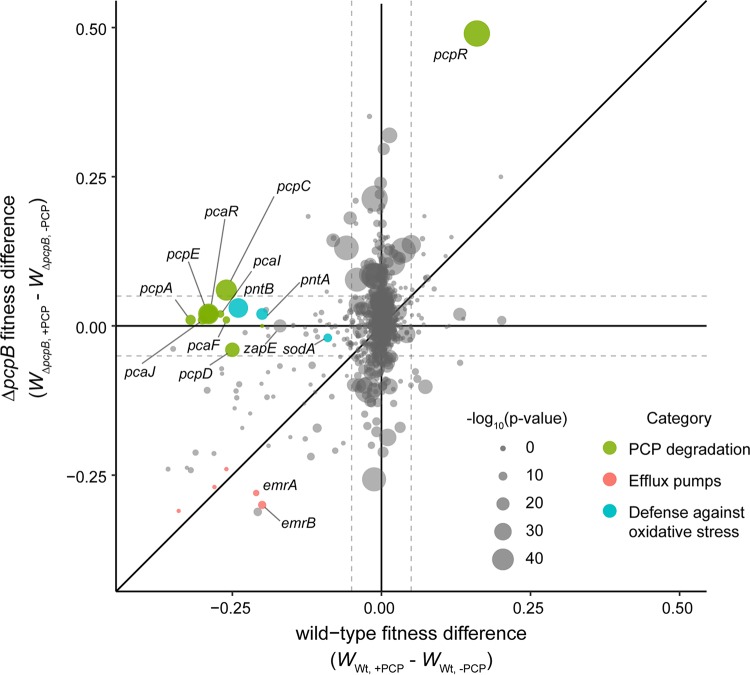
Comparison of the effect of gene disruption in wild-type and Δ*pcpB* S. chlorophenolicum as determined by Tn-seq. Fitness values (W) in the absence of PCP were subtracted from fitness values in the presence of PCP to remove the contribution to fitness due to gene disruption in the absence of PCP. A two-sample *t* test was used to test for significance between the fitness values in the presence of PCP between the wild-type and Δ*pcpB* strains. *P* values are indicated by the size of each data point. Dashed lines designate changes in fitness of plus or minus 0.05.

To distinguish between genes important for growth during PCP exposure and those important for growth during PCP degradation, we carried out the same Tn-seq experiment in the S. chlorophenolicum Δ*pcpB* strain (Data Set S1B), which cannot degrade PCP. The fitness effects of disrupting a given gene were reproducible between replicates (Fig. S5C and D). As expected, the importance of many genes for general defense against PCP, such as those encoding components of efflux pumps for the removal of PCP, was consistent between strains. We found a number of genes whose disruption impacted the Δ*pcpB* strain differently than the wild-type strain in the presence of PCP (Fig. 7). The Δ*pcpB* strain may experience higher stress in the presence of PCP (Fig. 2B) because it cannot degrade PCP once it enters the cell. Thus, disruption of a number of genes, including many whose disruption is insignificant in the wild-type strain, caused a more severe effect in the Δ*pcpB* strain.

The genes whose disruption was detrimental in the wild-type strain but not in the Δ*pcpB* strain were of particular interest, as these genes may be involved in processing the PCP metabolites and alleviating their toxic effects on the cell. As expected, disruption of genes encoding the PCP degradation enzymes and those involved in 3-oxoadipate metabolism compromised fitness in wild-type S. chlorophenolicum but not in the S. chlorophenolicum Δ*pcpB* strain. Disruption of only four other genes decreased fitness in this manner (Table 2). Three of these genes are expected to contribute to defense against ROS. Disruption of a gene encoding superoxide dismutase decreased fitness by 10% in the presence of PCP only in the wild-type strain. Disruption of *pntA* and *pntB*, which encode NAD(P)^+^ transhydrogenase, decreased fitness by 21% and 24%, respectively, in the presence of PCP only in the wild-type strain. NADPH is used to convert PCP to TCBQ and is required for reduction of glutathione disulfide, which is generated during reduction of TCHQ and TriCHQ by TCHQ dehalogenase. NADPH is also required for the reduction of H_2_O_2_ by glutathione peroxidase (51), as well as by reactions with superoxide (65), semiquinones, and benzoquinones (38, 66). The importance of *pntAB* and *sodA* only in a strain capable of degrading PCP suggests that increased levels of superoxide are produced during PCP degradation.

**TABLE 2:** Change in fitness caused by disruption of selected genes during PCP exposure^*a*^

Gene	Product	Δ fitness^*b*^ (*W*_Wt, +PCP_ – *W*_Wt, −PCP_) wild-type	Δ fitness^*b*^ (*W*_Δ*pcpB*, +PCP_ – *W*_Δ*pcpB*, −PCP_) *ΔpcpB*	*P* value^*c*^
***pcpR***	PCP transcriptional activator	0.16*	0.50*	4.8e−52
***pcpB***	PCP hydroxylase	−0.20*	n/a	n/a
***pcpD***	TCBQ reductase	−0.25*	-0.04	3.1e−23
***pcpC***	TCHQ dehalogenase	−0.25*	0.07*	1.2e−37
***pcpA***	DCHQ dioxygenase	−0.32*	0.01	3.0e−14
***pcpE***	Maleylacetate reductase	−0.29*	0.01	4.4e−31
***pcaJ***	3-Oxoacid CoA transferase, subunit B	−0.30*	0.01	2.0e−12
***pcaI***	3-Oxoacid CoA transferase, subunit A	−0.27*	0.01	2.6e−08
***pcaF***	Beta-ketoadipyl CoA thiolase	−0.26*	0.01	7.1e−08
***pcaR***	IclR family transcriptional regulator	−0.29*	0.02	1.2e−36
*sodA*	Superoxide dismutase	−0.10*	−0.02	9.8e−11
*pntA*	NAD(P)^+^ transhydrogenase, subunit A	−0.21*	0.02	2.0e−17
*pntB*	NAD(P)^+^ transhydrogenase, subunit B	−0.24*	0.03	3.5e−34
*zapE*	AFG1 family ATPase	−0.16*	0.00	1.2e−22

a Fitness values for all transposon integrants are listed in Data Set S1B. This table contains genes important during PCP degradation. Genes shown in bold type are upregulated after PCP exposure.

b Fitness (*W*) difference comparing the control and experimental (PCP-treated) conditions for each Tn-seq library. Significant fitness changes are those that cause a fitness difference of >0.05 and those for which *P* < 1.2e−5 (two-sample *t* test with a Bonferroni correction). Asterisks indicate significant differences.

c A two-sample *t* test was used to test for significant difference of disrupting a given gene between the PCP-treated wild-type and Δ*pcpB* Tn-seq libraries. n/a, not available.

## DISCUSSION

S. chlorophenolicum is exposed to a variety of stresses, including dissipation of the PMF, perturbation of the cell envelope, toxic alkylating agents, and ROS during degradation of PCP. Given the toxicity of the intermediates produced during PCP degradation, S. chlorophenolicum might be better off without degrading PCP. Resistance to PCP can be achieved by means other than degradation; some organisms detoxify PCP by methylating its hydroxyl group, resulting in formation of pentachloroanisole, which cannot dissipate the PMF (67). Microbes may simply exclude PCP from the cell due to an intrinsically impermeable cell envelope (68), by actively changing the permeability of the cell envelope (69), or as our data suggest, by using efflux pumps to expel PCP. However, complete degradation of PCP offers an obvious advantage in that it detoxifies the organism’s local environment and prevents the continuous expenditure of energy to power efflux pumps. It may also decrease the level of PCP that accumulates in the membrane by providing a cytoplasmic sink via the degradation pathway. In addition, PCP degradation allows the use of PCP as a source of carbon (Fig. 2C).

Despite the toxicity of the PCP degradation intermediates, the transcriptional response to PCP shows little overlap with those to methylglyoxal or paraquat, suggesting that the cells successfully alleviate stresses due to alkylating benzoquinones and ROS. The TCBQ produced by hydroxylation of PCP is sequestered at the active site of PcpB until it is reduced to the less toxic TCHQ by PcpD (39). However, the downstream chlorinated hydroquinone intermediates, which are present at a total concentration of approximately 61 µM (35), would be expected to generate ROS as well as reactive chlorinated benzoquinones. The problems generated by these intermediates are magnified by their potential to cause redox cycling, a process in which autoxidation of a hydroquinone produces superoxide and a benzoquinone, which can be reduced back to the hydroquinone, allowing the process to occur repeatedly. Disproportionation of superoxide by superoxide dismutase produces H_2_O and H_2_O_2_, which can form hydroxyl radicals via the Fe^+2^-catalyzed Fenton reaction. Although it is commonly assumed that TCHQ itself causes redox cycling, the chemistry of TCHQ, TriCHQ, and DCHQ *in vivo* is probably more complex. TCBQ produced by autoxidation of TCHQ reacts with thiols at a much higher rate than with NAD(P)H (70). Thus, chlorinated benzoquinones produced by autoxidation will preferentially react with glutathione in cells where glutathione levels are typically 10- to 100-fold higher than NAD(P)H levels (71). Thus, it is likely that the glutathione conjugates, rather than the chlorinated hydroquinones themselves, cause ROS production *in vivo*. The observation that disruption of the gene encoding superoxide dismutase is detrimental in wild-type S. chlorophenolicum, but not in the Δ*pcpB* strain, suggests that it plays an important role in protecting cells against ROS. Disruption of genes encoding glutathione peroxidase or either one of two different catalases is not detrimental. However, there may be sufficient redundancy conferred by these three H_2_O_2_-detoxifying enzymes that loss of any one does not affect fitness.

We discovered an interesting discrepancy between the effects of disruption of *pcpR* and *pcpB*. In both cases, no PCP degradation will occur. However, the Δ*pcpB* strain grows more slowly than the Δ*pcpR* strain does (Fig. 2) in PCP-depleted medium. Consistent with this observation, our Tn-seq analysis (where PCP concentrations are held constant) shows that disruption of *pcpR* increases growth rate by 16%, whereas disruption of *pcpB* decreases growth rate by 20% compared to wild-type S. chlorophenolicum exposed to PCP. Interestingly, deletion of *pcpR* in a Δ*pcpB* background appears to restore the fitness of the Δ*pcpB* strain (Table 2). These results suggest that PcpR may activate expression of other genes in addition to the PCP degradation genes themselves. If the PCP degradation pathway is evolutionarily derived from one or more pathways for degradation of naturally occurring phenols, then such a transcriptional regulator might activate expression of transporters that increase PCP import into the cytoplasm. When PCP cannot be degraded by the Δ*pcpB* strain, its functional PcpR would still increase expression of transporters that import PCP, thereby exacerbating perturbation of membrane fluidity and dissipation of the PMF. In support of this hypothesis, we observed that disruption of a gene encoding a membrane transporter (RS13025) was beneficial in the presence of PCP in the wild-type strain, and disruption of genes encoding four additional transporters was beneficial when PCP degradation was disabled by deletion of *pcpB* (Data Set S1D). If these transporters are indeed importing PCP, preventing their upregulation by deletion of *pcpR* may decrease the uptake of PCP into cells.

Our investigation into the response of S. chlorophenolicum to PCP exposure, the genes important for survival in the presence of PCP, and the stresses involved with PCP degradation provides insight into how this bacterium is able to accomplish complete mineralization of PCP using a pathway involving an unusual number of highly toxic intermediates. Further, our results suggest strategies that might be used for engineering a strain with improved capacity for degradation of PCP or other problematic phenolic biocides, either by adaptive laboratory evolution or targeted genetic changes. Our data indicate that loss of *pcpR* improves growth rate in rich medium; thus, attempts to use adaptive laboratory evolution to yield improved PCP degraders may select for cells that have simply lost the ability to degrade PCP unless PCP is required as a nutrient source (i.e., the medium contains low levels of other nutrients). Under low-nutrient conditions, mutations that increase flux through the PCP degradation pathway might improve both growth and degradation. If transporters are importing PCP and efflux pumps are exporting PCP (as discussed above), mutations that optimize the balance of PCP uptake and efflux could save unnecessary expenditure of energy while still providing sufficient PCP in the cytoplasm for entry into the degradation pathway. Mutations that alter outer membrane permeability might also optimize the balance between PCP uptake and efflux. Such adjustments might diminish dissipation of the PMF and dampen the massive global transcriptional response that wastes energy and resources. Useful targeted genetic manipulations might include deletion of genes such as those encoding the ECF σ-factor RS05130 or the transcriptional repressor *hrcA*, for both of which we found disruption to be beneficial. Overexpression of PntAB might also be helpful to increase production of NADPH for PCP metabolism and reduction of glutathione disulfide. Other studies have shown that overexpression of NADPH-producing machinery improves utilization of novel metabolic pathways (72). Additional strategies might include spatial recruitment of the PCP degradation enzymes to synthetic protein scaffolds (73), which may improve flux through the pathway. Finally, localization of the degradation enzymes to the inner membrane (74) might minimize diffusion of PCP as well as toxic intermediates throughout the cytoplasm.

## MATERIALS AND METHODS

### Strains and growth conditions.

Sphingobium chlorophenolicum L-1 (ATCC 53874) and Escherichia coli SM10 *λpir* were used in this work. E. coli was grown at 37˚C in Luria broth. S. chlorophenolicum was grown at 30˚C unless stated otherwise. S. chlorophenolicum was grown in 1/4-strength tryptic soy broth (1/4-TSB) for routine culture and RNA-seq experiments. Growth measurements were performed in a Thermo Electron Varioskan 3001 microplate reader. For Tn-seq, cultures were grown in S. chlorophenolicum defined (SCD) medium (3.7 mM K_2_HPO_4_, 1.4 mM KH_2_PO_4_, 0.4 mM MgSO_4_·7H_2_O, 5.9 mM NaNO_3_, 23.7 mM sodium glutamate, 20 µM FeSO_4_, 90 µM CaCl_2_ [pH 7.1] [75], and 0.1× EZ-Rich supplement mixes M2103 and M2104 [76] [which contain nucleobases, vitamins, and amino acids; threonine and leucine were omitted] [pH 7.1]). To evaluate growth on PCP as the sole carbon source, starter cultures grown in 1/4-TSB were used to inoculate minimal salts medium (3.7 mM K_2_HPO_4_, 1.4 mM KH_2_PO_4_, 0.4 mM MgSO_4_·7H_2_O, 5.9 mM NaNO_3_, 20 µM FeSO_4_, 90 µM CaCl_2_ [pH 7.1]) containing 200 µM PCP to an OD_600_ of 0.1. After incubation at 30˚C with shaking overnight, the cells were washed three times in minimal salts medium and used to inoculate minimal salts medium with and without 200 µM PCP to an initial OD_600_ of 0.001. Cultures were incubated with shaking at 30˚C for 8 days. Additional details are provided in Text S1 in the supplemental material.

### RNA-seq.

Total RNA was isolated from cultures after exposure to 200 µM PCP, 39 µM CCCP, 5 mM toluene, 20 µM paraquat, or 1 mM methylglyoxal for 15 min. cDNA libraries were constructed using the RNAtag-Seq protocol (77) and sequenced on an Illumina NextSeq500. Differential expression was determined with edgeR (v 3.20.6). RNA-seq results were validated with independently isolated RNA samples via RT-qPCR. Additional details are provided in Text S1.

### Tn-seq.

Mutant libraries were constructed using the *Himar1C* mariner transposase by conjugation with E. coli carrying pSAM_Sc (Fig. S4B) (78). Libraries were grown in the presence or absence of 200 µM PCP for 20 to 25 generations. Genomic DNA was extracted, and Tn-seq libraries were constructed as described by Wiles et al. (78) and sequenced on an Illumina NextSeq500. Data analysis was performed as described in the MaGenTa protocol (79) with minor modifications. Fitness results were confirmed by one-on-one competition assays between selected mutant strains and the wild-type strain. Mutant strains were constructed by introduction of a mutation cassette into target genes as described in Text S1. The mutant and wild-type strains were mixed in a 1:1 ratio and grown for 20 to 25 generations in the presence or absence of 200 µM PCP. Abundances of the two strains were measured by plating cultures on 1/4-TSB plates with and without the antibiotic to which the mutant strain is resistant (either kanamycin or hygromycin). Additional details are provided in Text S1.

### PMF measurements.

Intracellular pH changes were assessed using the ratiometric fluorescent probe 2′,7′-bis-(2-carboxyethyl)-5-(and-6)-carboxyfluorescein acetoxymethyl ester (BCECF-AM). Cells were incubated in 50 mM K_2_HPO_4_ (pH 6.5) containing 5 mM EDTA and 5 mM BCECF-AM for 1 h (80). Cells were washed three times in the same buffer, and fluorescence measurements were carried out in SCD medium on a Synergy H1 microplate reader at excitation/emission wavelengths of 455/535 nm (pH-independent fluorescence) and 490/535 nm (pH-dependent fluorescence).

### [ATP]/[ADP] measurements.

S. chlorophenolicum was exposed to 200 µM PCP, and cells were harvested after 15 min and 5 h. The relative concentrations of ATP and ADP were measuring using the EnzyLight ADP/ATP ratio assay kit (BioAssay Systems) according to the manufacturer’s instructions.

### Accession number(s).

Reads for RNA-seq and Tn-seq experiments were deposited to the NCBI GEO database under accession number GSE114149.
