# Supplementary figures and images for: Genome-Wide Analysis of Transcriptional Changes and Genes That Contribute to Fitness during Degradation of the Anthropogenic Pollutant Pentachlorophenol by Sphingobium chlorophenolicum
Source: mSystems. 2018 Nov 20;3(6):e00275-18. doi: 10.1128/mSystems.00275-18 (PMC6247019; doi:10.1128/mSystems.00275-18)

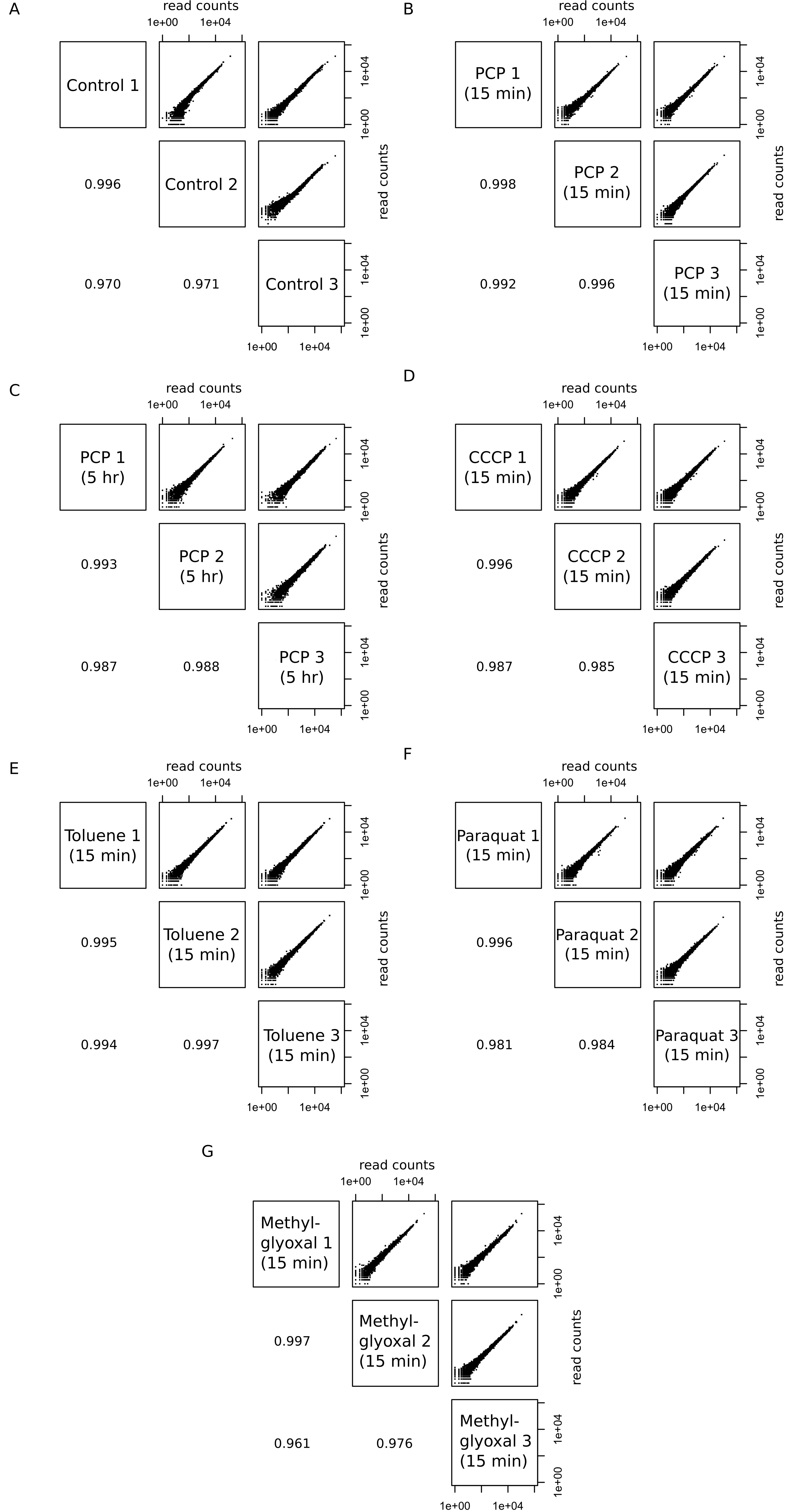

Supplement: FIG S1 [file sys006182294sf1.jpg]

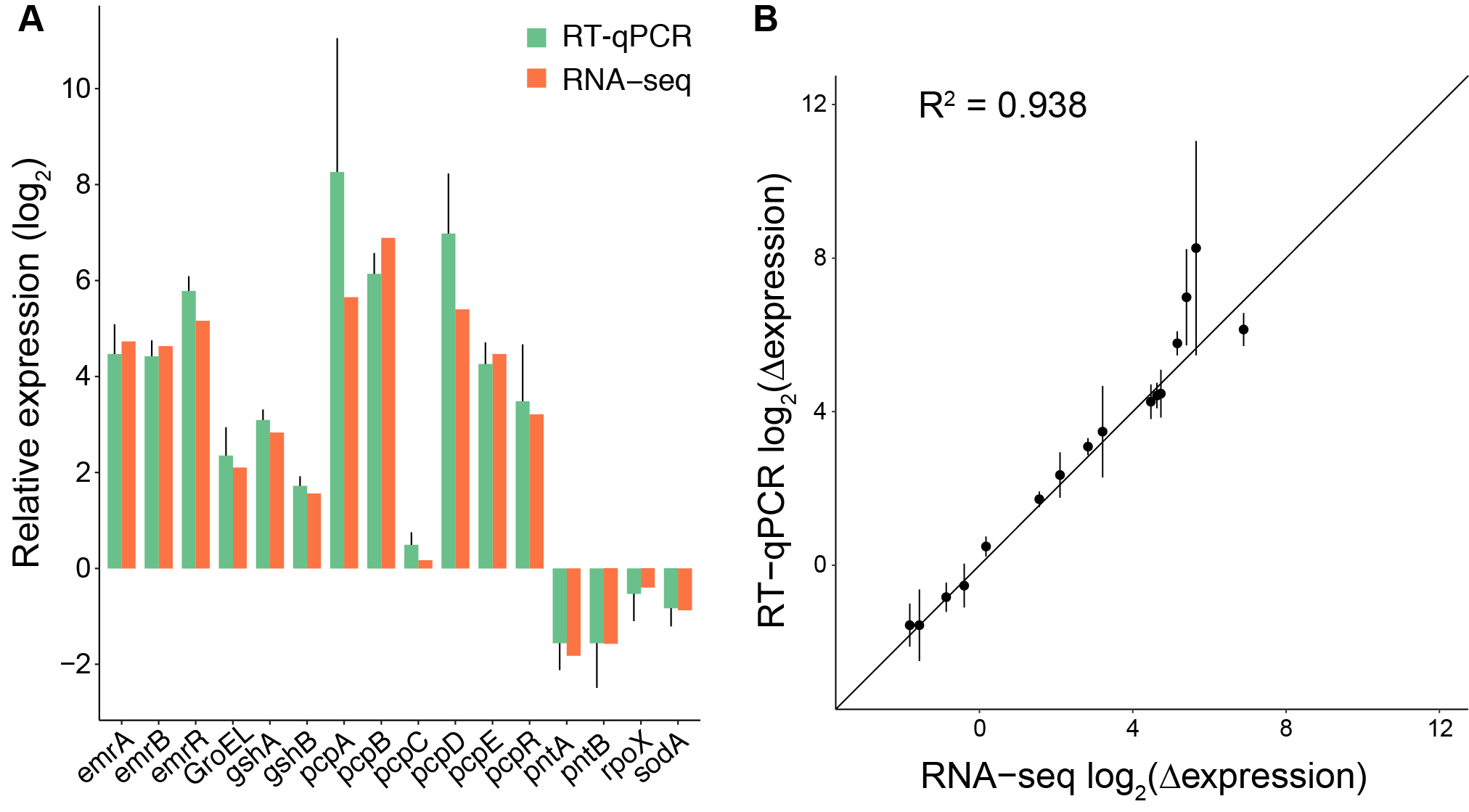

Supplement: FIG S2 [file sys006182294sf2.jpg]

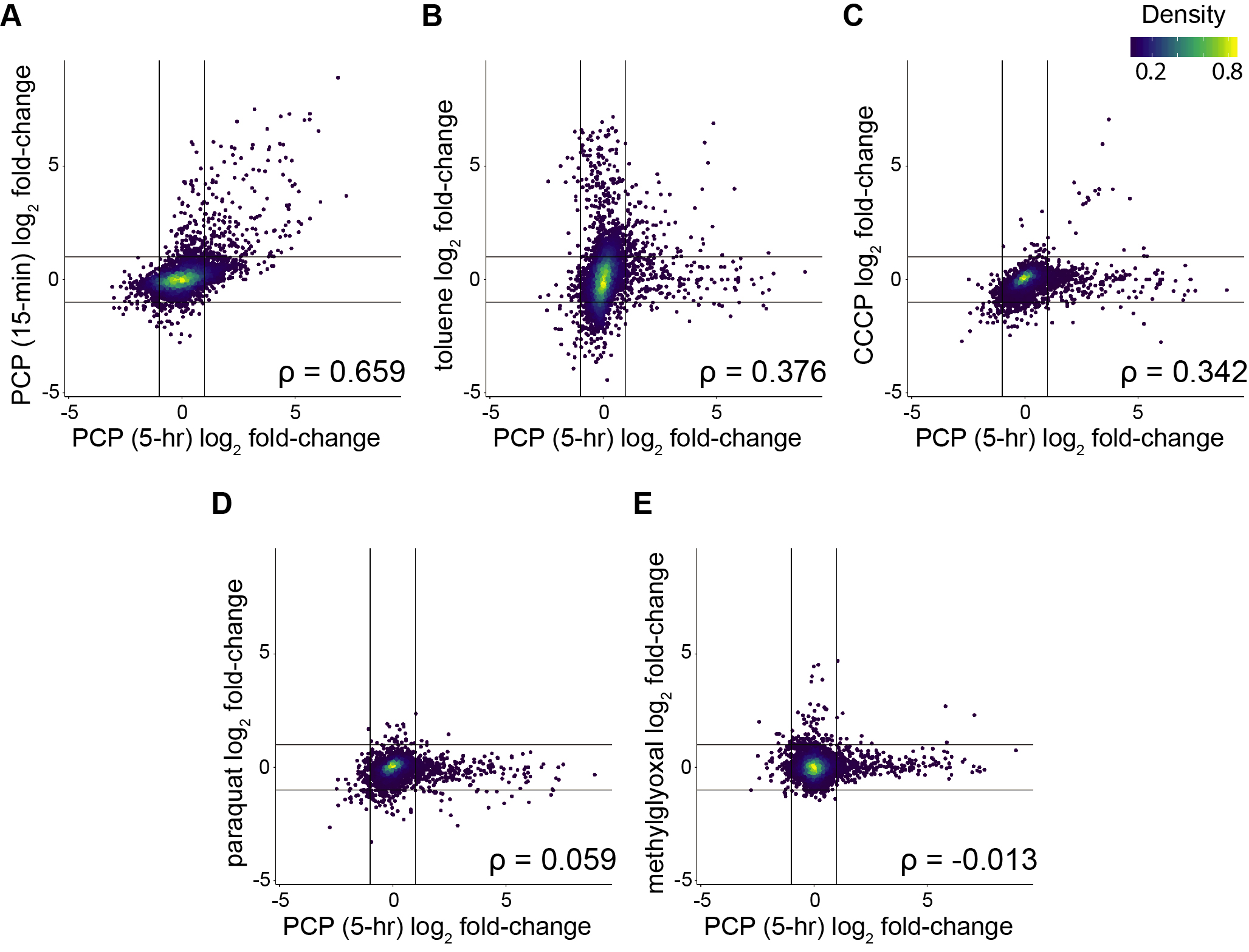

Supplement: FIG S3 [file sys006182294sf3.jpg]

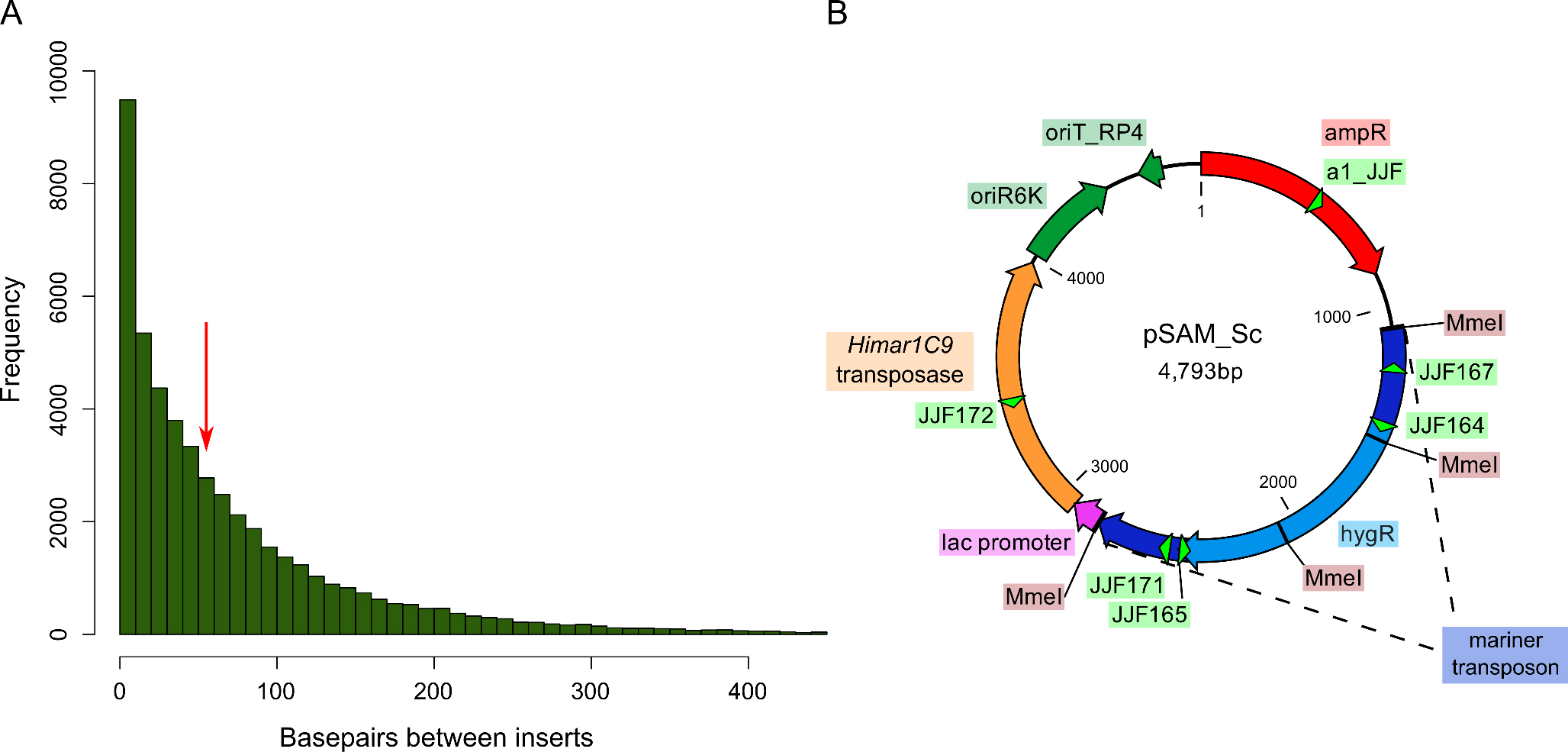

Supplement: FIG S4 [file sys006182294sf4.jpg]

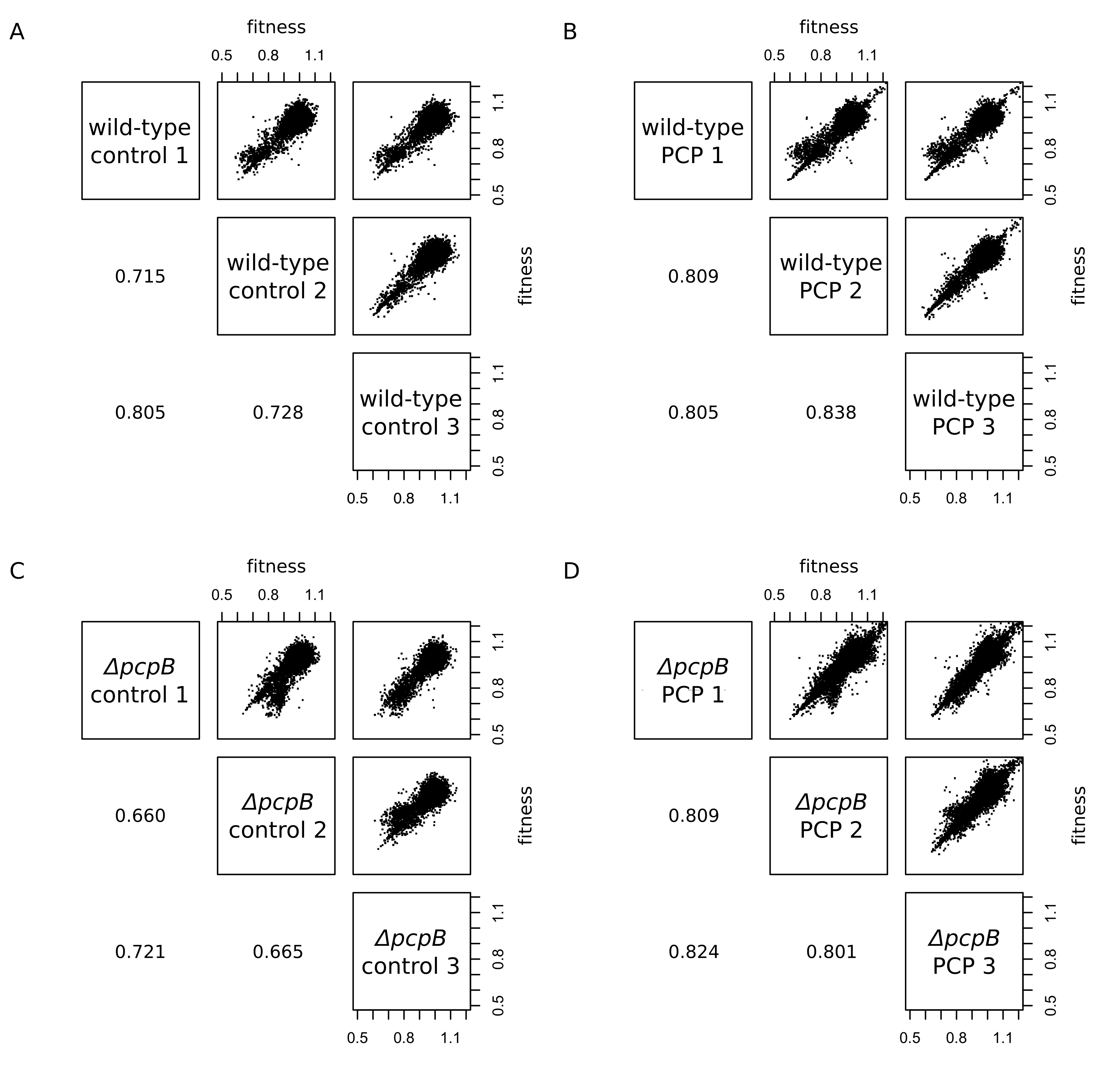

Supplement: FIG S5 [file sys006182294sf5.jpg]

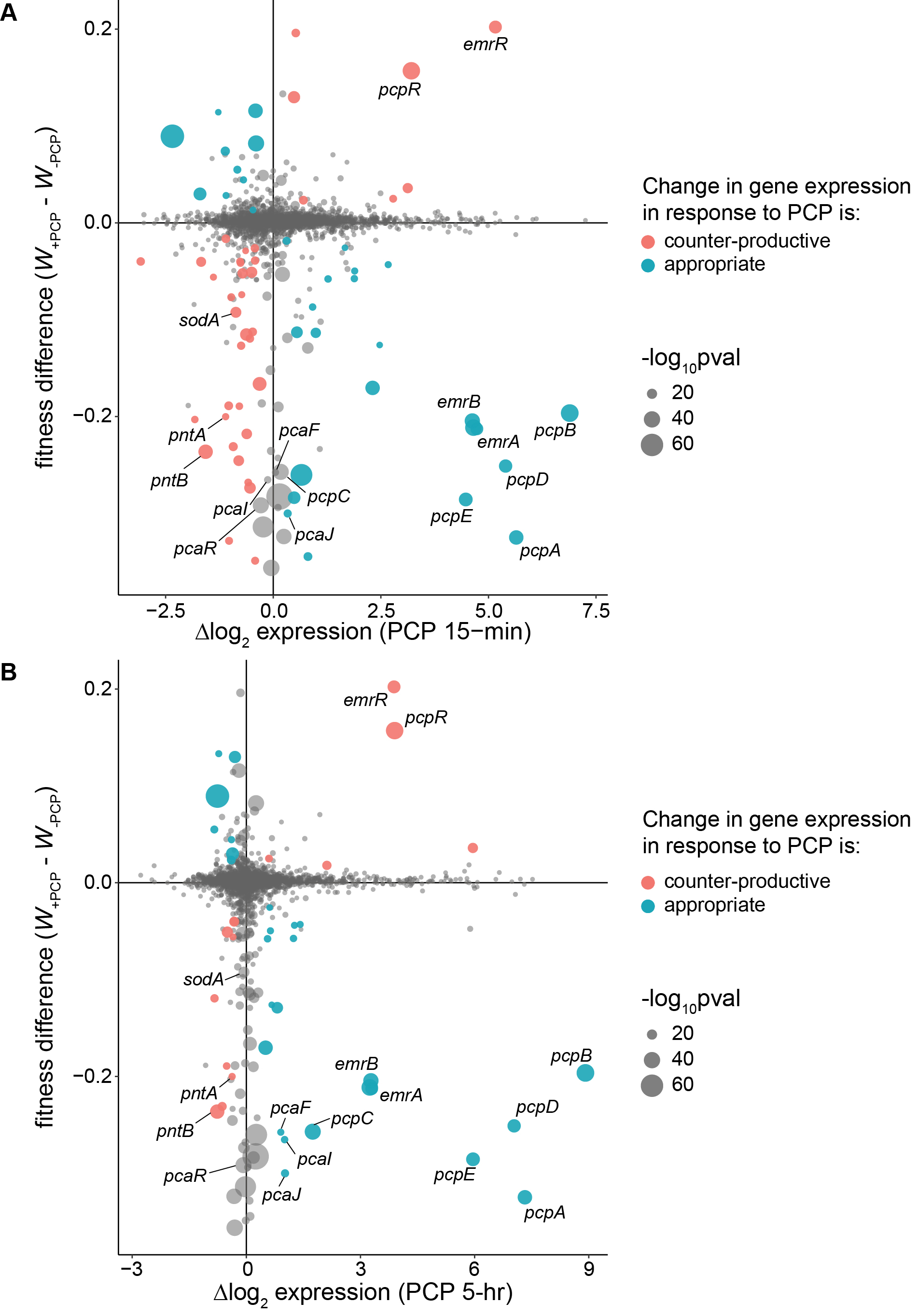

Supplement: FIG S6 [file sys006182294sf6.jpg]

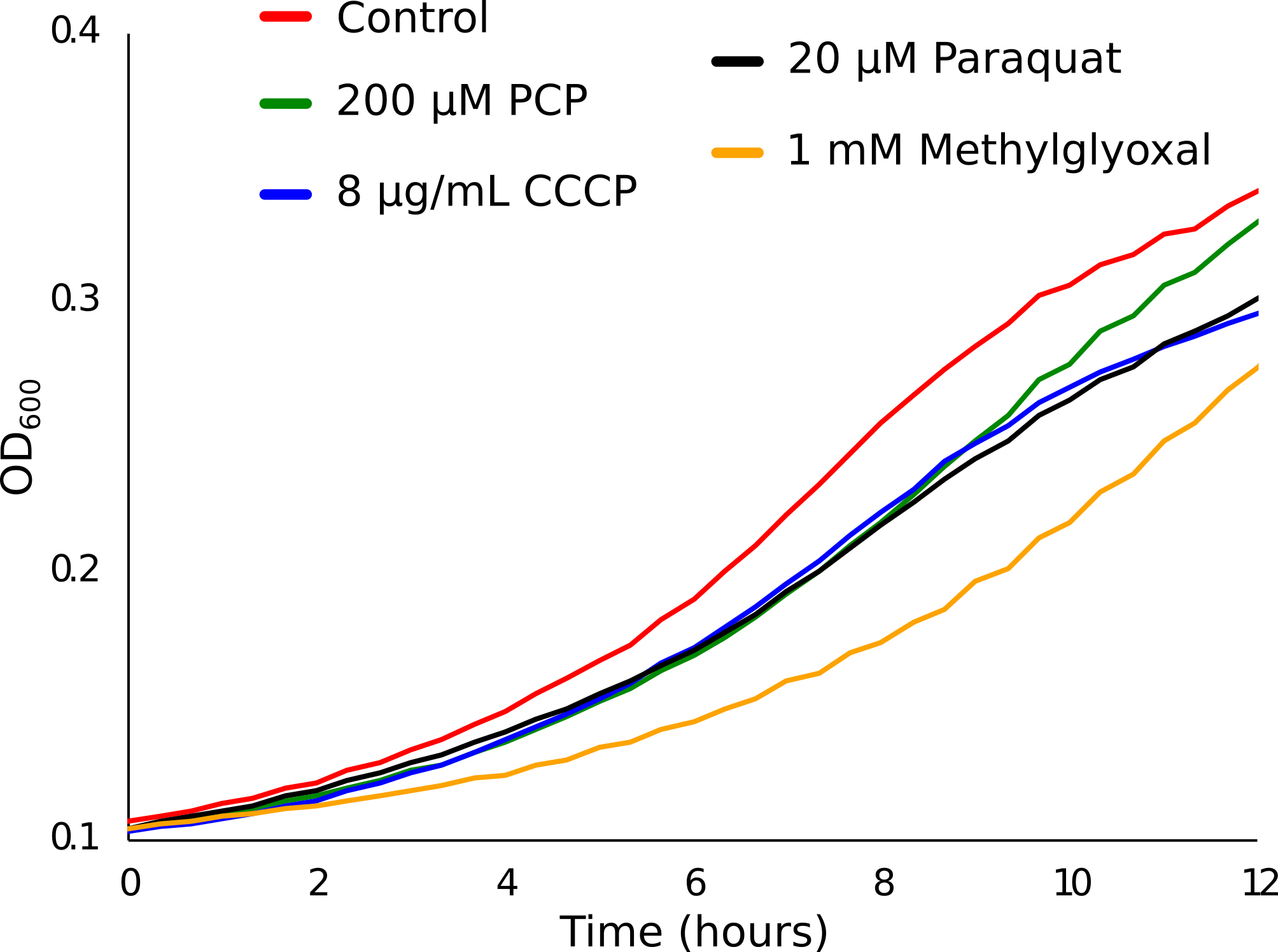

Supplement: FIG S7 [file sys006182294sf7.jpg]
