# Supplementary material for: Genome-Wide Analysis of Transcriptional Changes and Genes That Contribute to Fitness during Degradation of the Anthropogenic Pollutant Pentachlorophenol by Sphingobium chlorophenolicum
Source: mSystems. 2018 Nov 20;3(6):e00275-18. doi: 10.1128/mSystems.00275-18 (PMC6247019; doi:10.1128/mSystems.00275-18)
Supplement: TEXT S1 [file sys006182294s1.pdf]

Genome-wide analysis of transcriptional changes and genes that contribute to fitness during degradation of the anthropogenic pollutant pentachlorophenol by *S. chlorophenolicum*

Jake J. Flood[a,b], Shelley D. Copley[a,b]

[a]Department of Molecular, Cellular and Developmental Biology, University of Colorado Boulder, Boulder, Colorado, USA

[b]Cooperative Institute for Environmental Sciences, University of Colorado Boulder, Boulder, Colorado, USA

Supplementary Methods

Chemicals

Chemicals were obtained from the following suppliers: tryptic soy broth, EMD Millipore, #105459; Luria broth (LB), Alpha Biosciences, #L12-112; BCECF-AM, ThermoFisher, #B1170; methanol for HPLC, EMD Millipore, #MX0475. The following were obtained from Sigma Aldrich: carbonyl cyanide *m*-chlorophenyl hydrazine (CCCP) #C2759; paraquat, #856177; toluene, #179418; methylglyoxal, #M0252; pentachlorophenol, #P260-4. Primers were purchased from Integrated DNA technologies (IDT).

Growth of *S. chlorophenolicum*

For routine culture, an aliquot of a glycerol stock of *S. chlorophenolicum* L-1 (ATCC 53874) was streaked onto a ¼-strength tryptic soy broth (¼-TSB) agar plate and grown at 30˚C for 4-5 days. Colonies were used for cell cultures within 2 weeks. To start a liquid culture, a bent pipet tip was used to transfer several colonies into 25 mL ¼-TSB. The culture was grown at 30˚C with shaking overnight to saturation (OD_{600} 1.5-2.0). This starter culture was used to inoculate 25 mL of ¼-TSB to an OD_{600} of 0.01. The culture was grown at 30˚C with shaking until the desired OD_{600} was reached. (The doubling time for *S. chlorophenolicum* in ¼-TSB is about 3 hours.) *S. chlorophenolicum* was grown in ¼-TSB unless otherwise stated. Stocks of 200 mM PCP were made in DMSO, resulting in a DMSO concentration of 0.1% after dilution to give 200 µM PCP. All control cultures contained 0.1% DMSO unless stated otherwise. For Tn-Seq experiments, the transposon library was grown in ***S**. **c**hlorophenolicum* **d**efined (SCD) medium, which permits growth of *S. chlorophenolicum* transposon mutants in which the transposon has disrupted an otherwise essential biosynthesis gene, but prevents growth of *E. coli* SM10 *λpir*, which is auxotrophic for threonine and leucine. SCD medium (pH 7.1) contains 3.7 mM K_2HPO_4, 1.4 mM KH_2PO_4, 0.4 mM $MgSO_4 \cdot 7H_2O$, 5.9 mM $NaNO_3$, 23.7 mM sodium glutamate, 20 µM $FeSO_4$, 90 µM $CaCl_2$ (1), and 0.1x EZ-Rich supplement mixes M2103 and M2104 (2), which contain nucleobases, vitamins, and amino acids; threonine and leucine were omitted for reasons described in the Tn-seq section.

Deletion of genes in *S. chlorophenolicum*

Genes were deleted from the *S. chlorophenolicum* genome by homologous recombination with a suicide plasmid encoding resistance to hygromycin. Suicide plasmids were designed using NEBuilder (v1.12.18), with primers containing the appropriate overhanging regions to facilitate plasmid assembly. All PCR reactions were performed with Q5 Hot Start High-Fidelity PCR Master Mix (NEB #M0494S). PCR conditions for individual primer pairs are listed in Data S1E. Five-hundred basepair regions surrounding the target gene were amplified from genomic DNA. The upstream region was amplified using *gene*_HA1_F and *gene*_HA1_R and the downstream region was amplified using *gene*_HA2_F and *gene*_HA2_R, where "*gene"* is the gene of interest (Data S1E). The hygromycin resistance gene was amplified from pSAM_Sc using primers *gene*_hygR_F and *gene*_hygR_R (Data S1E), then digested with DpnI (NEB #R0176S) for 1 hour at 37˚C to remove template DNA. (DpnI digestion mixtures contained 30 µL DNA, 3.5 µL 10x Cutsmart buffer and 1 µL DpnI (20 U/mL)). The origin of replication for the suicide plasmid, which will prevent replication of the plasmid in *S. chlorophenolicum*, was amplified from pUC19 using primers pUC19_F and pUC19_R (Data S1E). All PCR fragments were run on a 0.8% agarose gel at 140V and purified with the Thermoscientific GeneJET Gel Extraction Kit (#K0691). These four fragments were then assembled via Gibson assembly in a reaction mixture containing 5 µL NEB Gibson Assembly Master Mix (#M5510A) and 0.125 pmol of each DNA fragment in a total volume of 10 µL. The reaction mixture was incubated at 50˚C for 60 minutes. The assembled plasmid was introduced into chemically competent *E. coli* 10G SOLO cells (Invitrogen #60106) by incubation of 25 µL cells with 2 µL of the plasmid on ice for 30

minutes, followed by incubation at 42˚C for 30 seconds. The cells were then immediately transferred back to ice to facilitate transformation. Cells were recovered in SOC medium (BD Difco #244310) for 1 hour at 37˚C with shaking, then plated on LB plates containing 100 µg/mL hygromycin and grown at 37˚C overnight. Colonies were used to inoculate 100 mL LB with 100 µg/mL hygromycin. These cultures were grown with shaking at 37˚C overnight and used to prepare mutation plasmid stocks using Qiagen Plasmid *Plus*Midi Kit (#12943). Plasmid yield was measured via Qubit dsDNA BR Assay Kit (Invitrogen #Q32850). Correct plasmid assembly and sequence were verified via Sanger Sequencing by Quintara Biosciences.

To introduce the desired mutation into the genome, a 500-mL culture of wild-type *S. chlorophenolicum* was grown to an OD_{600} of 0.4 as described above. Cells were rendered electrocompetent by washing three times in cold sterile 10% glycerol. Cells were resuspended in 10% glycerol to an OD_{600} of 100 and subjected to electroporation with 200-1000 ng of mutation plasmid that had been dialyzed on a 0.025 µm filter (Millipore VSWP01300) for 1 hour over 18-Ω water. Cells were allowed to recover for 24 hours in ¼-TSB with shaking at 30˚C and were then plated on ¼-TSB containing 5 µg/mL hygromycin to select for colonies in which the mutation cassette had been incorporated into the genome. Colonies grew after 5 days. For each transformation, 4 colonies were tested for successful integration of the mutation cassette via PCR using primers *gene*_oF and *gene*_oR (Data S1E). Sanger sequencing of the resulting fragment was carried out by Quintara Biosciences. All other mutants used in this study had been previously created as previously described (3).

RNA-Seq

<u>*Sample preparation*</u>

Cultures of *S. chlorophenolicum* were grown with shaking at 30˚C to an OD_{600} of 0.3-0.4 as described above. At that point, stressor compounds were added to give the indicated final concentrations: PCP, 200 μM; CCCP, 39 μM; toluene, 5 mM; paraquat, 20 μM; methylglyoxal, 1 mM. All stressors were dissolved in 0.41 mM NaOH at 1000x concentration before addition to cultures, resulting in a final concentration of 0.41 μM NaOH. NaOH was also added to the control cultures to give a final concentration of 0.41 μM. (All cultures were incubated with shaking at 30˚C for 15 minutes. Due to the slow growth rate of *S. chlorophenolicum*, little growth occurred during this time. For 5-hour PCP exposure, cultures were grown in ¼-TSB to an OD_{600} of 0.15. PCP was added to a final concentration of 200 μM and the cultures were incubated with shaking at 30˚C for 5 hours, by which time the OD_{600} had reached 0.3-0.4. (The concentration of PCP remained constant over this period.) All experiments were performed in triplicate. Cells were harvested by centrifugation at 4,000 x g and 4˚C for 8 minutes. The cell pellets were immediately treated with Qiagen RNAProtect Bacterial Reagent (#76506) as described by the manufacturer and subjected to centrifugation at 4,000 x g for 8 minutes at 4˚C. The supernatant was removed and the pellets were stored at -70˚C. RNA was purified using a Purelink RNA Mini Kit (Ambion, #12183020) and TURBO DNA-free kit (#AM1907). RNA quality was assessed on an agarose bleach gel (4). RNA-Seq libraries were prepared by the Biofrontiers Sequencing Facility at the University of Colorado Boulder following the RNAtag-Seq protocol (5). Briefly, purified RNA was fragmented to 200-300 base-pairs and the fragments were 5’-dephosphorylated and ligated to

barcoded RNA adaptors. Barcoded samples from different treatment conditions were pooled and ribosomal RNA was depleted with Ribo-zero (Illumina, #MRZMB126). cDNA libraries were prepared by reverse transcription, 3'-linker ligation, and amplification with indexed sequencing adaptors. The libraries were sequenced on an Illumina NextSeq500 at the University of Colorado Boulder Biofrontiers Sequencing Facility.

RNA-Seq data analysis

Raw sequencing data were de-multiplexed using fastx_barcode_splitter (11sep2008). Read quality was analyzed using the FastQC tool (v0.10.1). Read quality was high and no trimming was needed. The first 7 nucleotides (adaptor sequence) were removed with fastx_trimmer (v0.0.14). The reference genome sequence for *S. chlorophenolicum* L-1, Genbank assembly accession GCA_000147835.3, was downloaded from the NCBI Genome Database on June 27th, 2017. Reads were mapped to the *S. chlorophenolicum* genome using bowtie2 (v2.1.0) with default parameters. Sorting, indexing and file conversion were done with samtools (v0.1.19-96b5f2294a). Read counts in annotated regions of the genome were obtained using HTSeq (v0.7.2). Reads with alignment quality lower than 10 were discarded. Differential expression was analyzed with edgeR (v3.20.1) using default parameters. Unaligned and weakly expressed features (counts per million [CPM] < 1 in any sample replicate) were discarded.

Quantitative reverse-transcription PCR (RT-qPCR)

Preparation of cDNA

RNA-seq results were validated via RT-qPCR. RNA samples were prepared as described in the RNA-seq methods section from three replicate cultures that had been exposed to no stress (control) or to 200 μM PCP for 15 minutes. RNA was reverse transcribed using SuperScript IV VILO Master mix (ThermoFisher Scientific #11756050) according to the manufacturer's instructions. Briefly, 1.67 μg RNA was mixed with 8 μL master mix (either with or without reverse transcriptase (RT)) in a total volume of 40 μL. Reactions were incubated at 25˚C for 10 minutes, 50˚C for 10 minutes, then 85˚C for 5 minutes. The resulting cDNA was stored at -70˚C until use.

Primer design

Primers for qPCR were designed using the Primer-BLAST tool (6). Primer amplification efficiency was measured using genomic DNA from *S. chlorophenolicum* at the following concentrations: 1, 0.1, 0.01, 0.001, and 0.0001 ng/μL (Data S1E). Genomic DNA was isolated from cultures of wild-type *S. chlorophenolicum* using Invitrogen PureLink Genomic DNA Mini Kit (#K1820-02) according to the manufacturer's instructions. PCR reactions were carried out in a 384-well plate using the Eppendorf EpMotion 5070. Reactions contained 5 μL Power Sybr Green Master Mix (Applied Biosystems #4367659), 4 μL genomic DNA at the concentrations listed above, and 1 μL primers (final primer concentration: 2 μM). Reactions were run on a Biorad CFX384 Realtime System C1000 Thermocycler. (PCR conditions: initial denaturation, 95˚C for 10 minutes; 40 cycles of 95˚C for 15 sec, 60˚C for 60 sec, followed by a melt curve.) Amplification of the correct PCR product was confirmed by observation of a single peak

on the melt-curve profile, and by observation of a single band of the predicted size on a 0.8% agarose gel run at 140V.

qPCR

Quantitative PCR was performed on cDNA isolated as described above. Reactions contained 5 µL Power Sybr Green Master Mix (Applied Biosystems #4367659), 4 µL cDNA (1/50 dilution), and 1 µL primers (final primer concentration: 2 µM). qPCR was carried out as described in the "Primer Design" section above. Each sample was performed with 3 technical replicates for both RT and no-RT preparations. No-RT controls were used to eliminate the possibility of amplification due to contaminants such as genomic DNA. Expression levels were normalized to that of *recA*, whose expression does not change with PCP treatment. Gene fold changes were calculated taking into account the PCR amplification efficiencies (7):

$$Fold\ change = \frac{(E_{target})^{\Delta C_{q(target)}(control-sample)}}{(E_{ref})^{\Delta C_{q(ref)}(control-sample)}} \quad (1)$$

where E is amplification efficiency ($10^{(-1/slope)}$) and C_q is quantification cycle.

Modification of pSAM_Sc for transposon mutagenesis

pSAM_Ec (8) was modified to replace the kanamycin resistance gene with a hygromycin resistance gene, allowing generation of transposon libraries in both wild-type and Δ*pcpB::kan S. chlorophenolicum* (the latter of which has the kanamycin resistance gene used as a selection marker in pSAM_Ec). The backbone of pSAM_Ec, excluding the kanamycin resistance gene, was amplified with Q5 Hot Start High-Fidelity PCR Master Mix (NEB #M0494S) using primers JJF163 and JJF166. (PCR conditions: initial denaturation, 98 ˚C for 30 sec; 30 cycles of 98˚C for 10 sec, 61˚C for 30 sec, 72˚C

for 120 sec, followed by final elongation at 72˚C for 180 sec.) The hygromycin resistance gene (*hygR*) from plasmid YM48 (9) was amplified using primers JJF164 and JJF165. (PCR conditions: initial denaturation, 98˚C for 30 sec; 30 cycles of 98˚C for 10 sec, 61˚C for 30 sec; 72˚C for 120 sec, followed by final elongation at 72˚C for 180 sec.) The resulting fragments were then assembled using Gibson Assembly Master Mix (NEB, #M5510A) in reaction mixtures containing 5 µL NEB Gibson Assembly Master Mix (#M5510A), 20 fmol of pSAM_Ec backbone, and 50 fmol of *hygR* in a total volume of 10 µL. After incubation at 50˚C for 60 minutes, the assembled product was transformed into chemically competent *E. coli* MACH1 cells (ThermoFisher Scientific) (10). Successful cloning was confirmed by PCR amplification of the modified region of pSAM_Sc with primers a1_JJF and JJF165 (PCR conditions: initial denaturation, 98˚C for 30 sec; 30 cycles of 98˚C for 10 sec, 63˚C for 10 sec and 72˚C for 60 sec, followed by final elongation at 72˚C for 180 sec) and Sanger sequencing of the resulting fragment by Quintara Biosciences.

Tn-Seq

Creation of transposon library

Transposon libraries were created by introducing pSAM_Sc into *S. chlorophenolicum* by conjugation with *E. coli* SM10 *λpir* carrying plasmid pSAM_Sc (8, 11). Separate cultures of *S. chlorophenolicum* and *E. coli* carrying pSAM_Sc were grown to an OD_{600} of 0.8 in ¼-TSB (100 mL) and LB containing 100 µg/mL ampicillin (50 mL), respectively. Cultures were washed 3 times in cold, sterile phosphate-buffered saline (PBS), pH 7.4, containing 10 mM Na_2HPO_4, 1.8 mM KH_2PO_4, 137 mM NaCl and

2.7 mM KCl, mixed together in a 2:1 ratio of *S. chlorophenolium* to *E. coli*, and resuspended in 15 mL PBS. The cells were spotted onto SCD agar plates in 100 µL pools. After the spots had dried, the plates were incubated right-side up at 30˚C for 16 hours. *E. coli* SM10 *λpir* is auxotrophic for leucine and threonine, and therefore will not replicate on SCD-medium plates. After incubation, conjugation pools were scraped up and resuspended in 1 liter of SCD medium containing 10 µg/mL hygromycin. An aliquot was diluted and spread onto SCD agar plates with 10 µg/mL hygromycin to determine the number of unique transposon insertion events. The remainder of the culture was incubated with shaking at 30˚C for 3 days to an OD_{600} of 1.5 to select for clones that had integrated the transposon (encoding hygromycin resistance) into the genome. pSAM_Sc contains an RP4 oriT/oriR6K origin of replication and will only propagate in *pir*-positive strains. Thus, *S. chlorophenolicum* must express the *Himar1C* transposase and insert the transposon encoded on pSAM_Sc to gain hygromycin resistance. An aliquot of this culture was diluted and spread onto ¼-TSB plates with or without 10 µg/mL hygromycin and incubated at 30˚C for 4 days to determine library composition. An equivalent number of small, yellow colonies grew on plates with or without hygromycin, indicating that a majority of the culture contained successful transposon integrants. No large, white colonies were observed, indicating that the donor *E. coli* had not grown. Fifty mL of the remaining transposon library culture were centrifuged at 4,000 x g and 4˚C for 12 min. The pellet was resuspended in 5 mL SCD containing 20% glycerol, frozen in liquid nitrogen and stored at -70˚C.

PCR analyses with OneTaq Hot Start Master Mix (#M0484S) were used to confirm successful transposon integration into the *S. chlorophenolicum* genome.

Sixteen colonies from each transposon library were picked for analysis. Wild-type *S. chlorophenolicum* and *E. coli* SM10 *λpir* carrying pSAM_Sc were used as controls. The locations of the diagnostic primers used are indicated in Fig S5B. Primers JJF291 and JJF292, which amplify a region of the *S. chlorophenolicum* genome (SPHCH_RS19420), were used to confirm that the colonies were indeed *S. chlorophenolicum.* (PCR conditions: initial denaturation, 94˚C for 30 sec; 30 cycles of 94˚C for 15 sec, 44˚C for 30 sec, 68˚C for 60 sec, followed by final elongation at 68˚C for 300 sec.) Primers JJF164 and JJF165 were used to amplify the hygromycin resistance gene, confirming the presence of the *Himar1C* transposon. (PCR conditions: initial denaturation, 94˚C for 30 sec; 30 cycles of 94˚C for 15 sec, 44˚C for 30 sec, 68˚C for 60 sec, followed by final elongation at 68˚C for 300 sec.) Finally, primers a1_JJF and JJF167 and primers JJF171 and JJF172, which amplify regions of pSAM_Sc between the transposon and the plasmid backbone, were used to confirm that the plasmid backbone was no longer present, indicating that the transposon had been transferred from the plasmid to the genome. (PCR conditions for primers a1_JJF and JJF167: initial denaturation, 94˚C for 30 sec; 30 cycles of 94˚C for 15 sec, 51˚C for 30 sec, 68˚C for 60 sec, followed by final elongation at 68˚C for 300 sec. PCR conditions for primers JJF171 and JJF172: initial denaturation, 94˚C for 30 sec; 30 cycles of 94˚C for 15 sec, 49˚C for 30 sec, 68˚C for 60 sec, followed by final elongation at 68˚C for 300 sec.)

Transposon library outgrowth

A glycerol stock of each transposon library was thawed and used to inoculate 25 mL SCD medium with or without 200 µM PCP to an OD_{600} of 0.003. The control culture contained 0.1% DMSO, the carrier for the 200 mM stock PCP solution used for the

cultures in the presence of PCP. Each outgrowth was performed in triplicate. Every 8 hours, the OD_{600} was measured to assess cell growth and the PCP concentration was measured by HPLC. Cultures were diluted into fresh medium to an OD_{600} of 0.003 and PCP was added so that the final concentration was 200 µM, thereby maintaining the PCP concentration at 150-200 µM throughout the outgrowth. After each culture had grown for 20-25 generations, genomic DNA was isolated using the Purelink Genomic DNA Mini Kit (Invitrogen #K1820-02) according to the manufacturer's instructions. Genomic DNA was also purified from the transposon libraries before the outgrowth to serve as the t=0 sample.

Tn-Seq library construction

The transposon and the surrounding genomic DNA were isolated by digesting 7-14 µg DNA with a total of 18 U MmeI (NEB #R0637S) for 3 hours at 37˚C, adding fresh restriction enzyme every hour. MmeI cuts 18-20 base-pairs downstream of its recognition site, allowing us to isolate the transposon as well as the immediately surrounding genomic DNA. Entire digests were run on an 0.8% agarose gel at 140V. Because there is an MmeI cut site in the transposon itself, our desired fragments were 490 base-pairs and 752 base-pairs (Fig S5B). The region corresponding to 400-850 base-pairs fragments was cut out and DNA was purified with a GeneJET Gel Extraction Kit (Thermo Scientific K0692) according to the manufacturer's instructions, eluting in 30 µL water. The fragments were then dephosphorylated with Shrimp Alkaline Phosphatase (rSAP, NEB #M0371S) in a reaction mixture containing 29 µL DNA, 3.3 µL 10x Cutsmart buffer, and 1 µL rSAP. The reaction mixture was incubated at 37˚C for 30 min and then 65˚C for 5 min. Next, custom double-stranded Tn-seq adaptors

synthesized using primers tnseq005 and tnseq006 as described previously (11) were ligated onto the transposon fragments in a 1:16 molar ratio of DNA:adaptor using T4 DNA ligase (NEB #M0202S) in a reaction mixture containing 33.3 µL dephosphoryated DNA, 32 pmol Tn-seq adaptor, 1 mM ATP, and 2 µL ligase in a total volume of 46 µL. The reaction mixture was incubated overnight at 16˚C. The samples were purified using AMPure XP beads (#A63880) according to the manufacturer's instructions and amplified using primers Truseq_unv_adaptor and JJF162 with Q5 Hot Start High-Fidelity PCR Master Mix (NEB #M0494S). (PCR conditions: initial denaturation, 98˚C for 30 sec; 10 cycles of 98˚C for 10 sec, 72˚C for 40 sec, followed by final elongation at 72˚C for 60 sec.) The samples were purified with AMPure XP beads and amplified using sample-specific primers Seq004_TNseq_JJF and NEBNext index primers (E7335S & E7500S) using the same conditions described for the previous PCR. The samples were purified with AMPure XP beads and sequenced on an Illumina NextSeq500 at the Biofrontiers Sequencing Facility at the University of Colorado, Boulder.

Tn-Seq data analysis

After de-multiplexing using fastx_barcode_splitter (11sep2008), initial read quality was assessed using fastqc (v0.10.1). Reads were trimmed to 27 base-pairs from the 3' end using fastx_trimmer (v0.0.14). Because MmeI cuts at a variable distance (20-21 base-pairs) from its recognition site (12), reads were separated by the distance between the transposon and the cut site using fastx_barcode_splitter. Remaining transposon DNA was trimmed from the reads, leaving 15-17 base-pairs of genomic DNA to identify the position of the transposon. Reads with a Phred score < 30 in more than 50% of the read were removed using fastq_quality_filter (v0.0.14). Samtools

(v0.1.19) was used to convert files for visualization in Savant (v2.0.4). The reference genome sequence for *S. chlorophenolicum* L-1, Genbank assembly accession GCA_000147835.3, was downloaded from the NCBI Genome Database on June 27th, 2017. Reads were mapped to the *S. chlorophenolicum* genome with bowtie (v1.0.0) using options "--best" and "--tryhard". Maximum seed mismatch was set to 1. Reads aligning to multiple places in the genome were discarded, as there is no way to identify which gene has been disrupted. Samples were then analyzed as described in the Microbial Assessment by Genome-Wide Tn-Seq Analysis (MAGenTA) manual (13, 14) using default parameters except where described below. Fitness values were calculated using MAGenTA calc_fitness.py. Inserts with fewer than 8 reads and inserts occurring in the first or last 5% of the gene were eliminated from the analysis. Inserts that were present before outgrowth (t=0) but were completely absent after outgrowth were assigned one read for the outgrowth sample so that more accurate fitness values could be calculated. Fitness values for each individual insertion were calculated as previously described (14) by comparing the fold change of the mutant relative to the rest of the population, taking into account the number of generations the population was grown, with the following equation (15, 16):

$$W_i = \frac{\ln\left(N_i(t_2) \times \frac{2^d}{N_i(t_1)}\right)}{\ln\left(\left(1 - N_i(t_2)\right) \times \frac{2^d}{1 - N_i(t_1)}\right)} \tag{2}$$

where W_i is fitness and $N_i(t_1)$ and $N_i(t_2)$ are the frequencies of the mutant before and after outgrowth, respectively, and *d* is the number of generations during the outgrowth. Fitness values across each gene and across biological replicates were averaged to obtain a single fitness value associated with disruption of that gene using MAGenTA

aggregate.py. To determine significant fitness differences due to gene disruption, we performed a one-sample t-test against a population mean of 1 (neutral fitness). To determine significant fitness differences after gene disruption between two different conditions, we performed a two-sample t-test. Disruption of a gene was considered significantly detrimental or beneficial when (i) fitness values differed by >0.05 and (ii) p-values resulting from the t-test were <0.000012 (Bonferroni corrected p-value cutoff: 0.05 / # comparisons). Genes longer than 400 base-pairs with fewer than 4 insertions were considered lethal, while those shorter than 400 base-pairs with fewer than 4 insertions were deemed to lack sufficient information for a fitness category assignment ("insufficient info").

Competition assays

To validate the Tn-seq fitness measures, mutant strains were grown in competition with wild-type *S. chlorophenolicum* under the same conditions used in the Tn-seq outgrowth. First, cultures of wild-type and mutant strains were grown to an OD_{600} of 0.4-0.7 in SCD medium as described above and used to inoculate 2 mL SCD medium with or without 200 µM PCP in a 1:1 ratio of wild-type to mutant to give a final OD_{600} of 0.003. These cultures were grown with shaking at 30˚C. As described in the Tn-seq outgrowth, cultures were passaged every 12 hours into fresh SCD medium with or without PCP to an OD_{600} of 0.003. The number of generations per culture was tracked by measuring the OD_{600} before each passage. All cultures were grown for 20-25 generations. Competition cultures were diluted and plated onto ¼-TSB plates or ¼-TSB plates containing either 5 µg/mL kanamycin or 5 µg/mL hygromycin (depending on the

mutant, see Table S1) immediately after mixing wild-type and mutant (t=0) and after completing each outgrowth. Dilutions were made so that plates contained 30-300 colony forming units (CFU). Plates were grown at 22˚C for 5-7 days until colonies appeared, then used for fitness calculations as described in the Tn-seq methods section. Every competition assay was repeated in biological triplicate. Every culture was plated three times for technical replicates.

Measurement of PCP concentrations

PCP concentrations in cultures of *S. chlorophenolicum* were measured by high-performance liquid chromatography using an Agilent 1100 Series HPLC. Cultures were subjected to centrifugation at 16,000 x g for 1 minute at room temperature to remove particulates. Aliquots of the supernatant were injected onto a Zorbax SB-C18 (4.6 x 150 mm) column (PN: 883975-902) using a mobile phase of 70% methanol/ 30% H_2O at a flow rate of 1 mL/min. Absorbance was measured at 320 nm. PCP eluted at a retention time of 2.5 minutes.

Supplemental References

1. Pfennig N. 1967. Photosynthetic bacteria. Annu Rev Microbiol 21:285–324.
2. Neidhardt FC, Bloch PL, Smith DF. 1974. Culture Medium for Enterobacteria. J Bacteriol 119:736–747.
3. Yadid I, Rudolph J, Hlouchova K, Copley SD. 2013. Sequestration of a highly reactive intermediate in an evolving pathway for degradation of

pentachlorophenol. Proc Natl Acad Sci U S A 110:E2182-90.

4. Aranda PS, LaJoie DM, Jorcyk CL. 2012. Bleach gel: A simple agarose gel for analyzing RNA quality. Electrophoresis 33:366–369.

5. Shishkin AA, Giannoukos G, Kucukural A, Ciulla D, Busby M, Surka C, Chen J, Bhattacharyya RP, Rudy RF, Patel M, Novod N, Hung DT, Gnirke A, Garber M, Guttman M, Livny J. 2015. Simultaneous generation of many RNA-seq libraries in a single reaction. Nat Methods 12:323–325.

6. Coulouris Y, Zaretskaya I, Cutcutache I, Rozen S, Madden T. 2012. Primer-BLAST: A tool to design target-specific primers for polymerse chain reaction. BMC Bioinformatics 18:13:134.

7. Pfaffl MW. 2001. A new mathematical model for relative quantification in real-time RT-PCR. Nucleic Acids Res 29:45e–45.

8. Wiles TJ, Norton JP, Russell CW, Dalley BK, Fischer KF, Mulvey MA. 2013. Combining quantitative genetic footprinting and trait enrichment analysis to identify fitness determinants of a bacterial pathogen. PLoS Genet 9.

9. Janke C, Magiera MM, Rathfelder N, Taxis C, Reber S, Maekawa H, Moreno-Borchart A, Doenges G, Schwob E, Schiebel E, Knop M. 2004. A versatile toolbox for PCR-based tagging of yeast genes: New fluorescent proteins, more markers and promoter substitution cassettes. Yeast 21:947–962.

10. Sambrook J, Russell DW. 2000. Molecular cloning, a laboratory manual, 3rd ed. Cold Spring Harbor Laboratory Press.

11. Goodman AL, McNulty NP, Zhao Y, Leip D, Mitra RD, Lozupone CA, Knight R, Gordon JI. 2009. Identifying genetic determinants needed to establish a human

gut symbiont in Its habitat. Cell Host Microbe 6:279–289.

12. Emmersen J, Heidenblut AM, Hogh AL, Hahn SA, Welinder KG, Nielsen KL. 2007. Discarding duplicate ditags in LongSAGE analysis may introduce significant error. BMC Bioinformatics 8:1–12.

13. McCoy KM, Antonio ML, van Opijnen T. 2017. MAGenTA - Microbial Assessment by Genome-Wide Tn-Seq Analysis - Manual. Boston College, Chestnut Hill, MA.

14. van Opijnen T, Bodi KL, Camilli A. 2009. Tn-seq: high-throughput parallel sequencing for fitness and genetic interaction studies in microorganisms. Nat Methods 6:767–72.

15. Lenski RE, Rose MR, Simpson SC, Tadler SC. 1991. Long-Term Experimental Evolution in Escherichia coli. I. Adaptation and Divergence During 2,000 Generations 138:1315–1341.

16. van Opijnen T, Boerlijst MC, Berkhout B. 2006. Effects of Random Mutations in the Human Immunodeficiency Virus Type 1 Transcriptional Promoter on Viral Fitness in Different Host Cell Environments. J Virol 80:6678–6685.
