# Supplementary material for: Genome-Wide Analysis of Transcriptional Changes and Genes That Contribute to Fitness during Degradation of the Anthropogenic Pollutant Pentachlorophenol by Sphingobium chlorophenolicum
Source: mSystems. 2018 Nov 20;3(6):e00275-18. doi: 10.1128/mSystems.00275-18 (PMC6247019; doi:10.1128/mSystems.00275-18)
Supplement: TABLE S1 [file sys006182294st1.pdf]

Table S1. Validation of Tn-seq results by growth competition assays.

Strain[a]	Condition	Fitness (competition assay)[b]	Fitness (Tn-seq)[b]
Δ*pcpR::kanR*	control	1.00 +/- 0.009	1.00 +/- 0.017
Δ*pcpB::kanR*	control	1.04 +/- 0.02	1.00 +/- 0.013
ΔRS17200*::kanR*	control	0.97 +/- 0.02	1.00 +/- 0.014
Δ*pntA::hygR*	control	0.97 +/- 0.01	0.96 +/- 0.022
Δ*RS04025::hygR*	control	0.78 +/- 0.07	0.83 +/- 0.031
Δ*RS05130::hygR*	control	1.02 +/- 0.03	1.02 +/- 0.009
Δ*RS13025::hygR*	control	1.00 +/- 0.02	1.00 +/- 0.01
Δ*pcpR::kanR*	PCP	1.23 +/- 0.088	1.16 +/- 0.013
Δ*pcpB::kanR*	PCP	0.84 +/- 0.06	0.80 +/- 0.051
ΔRS17200*::kanR*	PCP	0.93 +/- 0.05	1.02 +/- 0.012
Δ*pntA::hygR*	PCP	0.83 +/- 0.05	0.75 +/- 0.052
Δ*RS04025::hygR*	PCP	0.82 +/- 0.03	0.78 +/- 0.024
ΔRS05130*::hygR*	PCP	1.25 +/- 0.02	1.11 +/- 0.009
ΔRS13025*::hygR*	PCP	1.12 +/- 0.06	1.06 +/- 0.01

[a] Antibiotic resistance used for competition assay is indicated in parenthesis
[b] Error represents standard deviation
